# Unsupervised Bayesian linear unmixing of gene expression microarrays

**DOI:** 10.1186/1471-2105-14-99

**Published:** 2013-03-19

**Authors:** Cécile Bazot, Nicolas Dobigeon, Jean-Yves Tourneret, Aimee K Zaas, Geoffrey S Ginsburg, Alfred O Hero III

**Affiliations:** 1 University of Toulouse, IRIT/INP-ENSEEIHT, 2 rue Camichel, 31071 Toulouse cedex 7, BP 7122, France; 2 Department of Medicine and Institute for Genome Sciences and Policy, Duke University, Durham, North Carolina, USA; 3 Center for Computational Biology and Bioinformatics and EECS Department, University of Michigan, 1301 Beal Avenue, Ann Arbor, MI, 48109-2122, USA

## Abstract

**Background:**

This paper introduces a new constrained model and the corresponding algorithm, called unsupervised Bayesian linear unmixing (uBLU), to identify biological signatures from high dimensional assays like gene expression microarrays. The basis for uBLU is a Bayesian model for the data samples which are represented as an additive mixture of random positive gene signatures, called *factors*, with random positive mixing coefficients, called *factor scores*, that specify the relative contribution of each signature to a specific sample. The particularity of the proposed method is that uBLU constrains the factor loadings to be non-negative and the factor scores to be probability distributions over the factors. Furthermore, it also provides estimates of the number of factors. A Gibbs sampling strategy is adopted here to generate random samples according to the posterior distribution of the factors, factor scores, and number of factors. These samples are then used to estimate all the unknown parameters.

**Results:**

Firstly, the proposed uBLU method is applied to several simulated datasets with known ground truth and compared with previous factor decomposition methods, such as principal component analysis (PCA), non negative matrix factorization (NMF), Bayesian factor regression modeling (BFRM), and the gradient-based algorithm for general matrix factorization (GB-GMF). Secondly, we illustrate the application of uBLU on a real time-evolving gene expression dataset from a recent viral challenge study in which individuals have been inoculated with influenza A/H3N2/Wisconsin. We show that the uBLU method significantly outperforms the other methods on the simulated and real data sets considered here.

**Conclusions:**

The results obtained on synthetic and real data illustrate the accuracy of the proposed uBLU method when compared to other factor decomposition methods from the literature (PCA, NMF, BFRM, and GB-GMF). The uBLU method identifies an inflammatory component closely associated with clinical symptom scores collected during the study. Using a constrained model allows recovery of all the inflammatory genes in a single factor.

## Background

Factor analysis methods such as principal component analysis (PCA) have been widely studied and can be used for discovering the patterns of differential expression in time course and/or multiple treatment biological experiments using gene or protein microarray samples. These methods aim at finding a decomposition of the observation matrix $Y\in R^{G\times N}$ whose rows (respectively columns) are indexed by gene index (respectively sample index). Typically, in gene expression analysis, the number *N* of samples is much less than the number *G* of genes. For example, in an Affymetrix HU133 gene chip, the number *G* of genes may range from ten to twenty thousand depending on the type of chip description file (CDF) processing used to translate the oligonucleotide fragments to gene labels whereas we only analyze about a hundred of samples.

This decomposition expresses each of the *N* samples as a particular linear combination of *R* characteristic gene expression signatures, also referred to as *factors*, with appropriate proportions (or contributions), called *factor scores*, following a linear mixing model

(1) Y=MA+N

where $M\in R^{G\times R}$ represents the *factor loading* matrix, $A\in R^{R\times N}$ the factor score matrix and $N\in R^{G\times N}$ is a matrix containing noise samples. Each sample **y**_*i*_ (*i* = 1, …, *N*), corresponding to the *i*-th column of the observed gene expression matrix **Y**, is a vector of *G* gene expression levels that can be expressed as

(2) $$y_{i}=\overset{R}{\underset{r=1}{\sum }}m_{r}a_{r,i}+n_{i}={\mathrm{Ma}}_{i}+n_{i}$$

where **m**_*r*_ is the *r*-th column of **M**, *a*_*r*, *i*_ denotes the (*r*, *i*)-th element of the matrix **A**, **a**_*i*_ and **n**_*i*_ are the *i*-th column of **A** and **N** respectively. The number of factors *R* in the decomposition is usually much less than the number of samples *N*. Traditional factor analysis methods such as PCA require the experimenter to specify the desired number of factors to be estimated. However, some recent Bayesian factor analysis methods are totally unsupervised in the sense that the number of factors is directly estimated from the data [1-3].

The model (1) is identical to the standard factor analysis model [4] for which the columns of **M** are called *factors* and should correspond to biological signatures (or pathways). Note that the elements of the matrix **M** are referred to as *factor loadings*, and the columns of **A** are the *factor scores*. Approaches to fitting the model (1) to data include principal component analysis [5,6], least squares matrix factorization [7,8], finite mixture modeling [9,10], and Bayesian factor analysis [4,11,12].

This paper presents a new Bayesian factor analysis method called unsupervised Bayesian linear unmixing (uBLU), that estimates the number of factors and incorporates non-negativity constraints on the factors and factor scores, as well as a sum-to-one constraint for the factor scores. The uBLU method presented here differs from the BLU method, developed in [13] for hyperspectral imaging and applied to gene microarray expression analysis in [14]. Note that BLU requires user specification of the number of factors while uBLU determines the number of factors using Bayesian birth-death model. The positivity and sum-to-one constraints are natural in gene microarray analysis when the entries of the observation matrix are non-negative and when a proportional decomposition is desired. Thus each factor score corresponds to the concentration (or proportion) of a particular factor to a given sample. The advantage of this representation for gene expression analysis is twofold: i) the factor scores correspond to the strengths of each gene contributing to that factor; ii) for each gene chip the factor scores give the relative abundance of each factor present in the chip. For example, a gene having a large loading level (close to one) for a particular factor should have a small loading (close to zero) for all other factors. In this way, as opposed to other factor analysis methods, there is less multiplexing making it easier to associate specific genes to specific factors and vice versa.

A similar approach, based on NMR spectral imaging and called the *Bayesian decomposition* (BD), has been previously developed by Moloshok *et al.* and applied to gene expression data [11]. More recently, the coordinated gene activity in pattern sets method (CoGAPS), available as an open R-source [12], combines the GAPS-MCMC matrix factorization algorithm with a threshold-independent statistic to infer activity in specific gene sets. However, these approaches require cold restarts of the algorithm with different number of factors and with different random seeds to avoid the large number of local minima encountered in the process of fitting the matrix factorization model **MA** to the data **Y**. In contrast, the proposed uBLU algorithm uses a judicious model to reduce sensitivity to local minima rather than using cold restarts. The novelty of the uBLU model is that it consists of: (1) a birth-death process to infer the number of factors; (2) a positivity constraint on the loading and score matrices **M**, **A** to restrict the space of solutions; (3) a sum-to-one constraint on the columns of **A** to further restrict the solution space. The uBLU model is justified for non-negative data problems like gene expression analysis and produces an estimate of the non-negative factors in addition to their proportional representation in each sample.

Bayesian linear unmixing, traditionally used for hyperspectral image analysis (see [13] for example), is one of many possible factor analysis methods that could be applied to gene expression analysis. These methods include non-negative matrix factorization (NMF) [7,8], independent component analysis (ICA) [15], Bayesian decomposition (BD) [11], PCA [5], bi-clustering [16], penalized matrix decomposition (PMD) [2], Bayesian factor regression modeling (BFRM) [1], and more recently the gradient-based algorithm of Nikulin *et al.* for general matrix factorization (GB-GMF) [17]. Contrary to uBLU, the PCA, ICA, BFRM, GB-GMF, bi-clustering and PMD methods do not account for non-negativity of the factor loadings and factor scores. On the other hand, NMF does not account for sum-to-one constraints on the columns of the factor score matrix. Contrary to PCA and ICA, uBLU does not impose orthogonality or independence on the factors, as well as the GB-GMF algorithm. These relaxed assumptions might better represent what is known about the preponderance of overlap and dependency in biological pathways. Finally, uBLU naturally accommodates Bayesian estimation of the number of factors, like BFRM. Note that BFRM has been specifically developed for gene expression data [1].

In this paper we provide comparative studies that establish quantitative performance advantages of the proposed constrained model and its corresponding uBLU algorithm with respect to PCA, NMF, BFRM and GB-GMF for time-varying gene expression analysis, using synthetic data with known ground truth. We also illustrate the application of uBLU to the analysis of a real gene expression dataset from a recent viral challenge study [18] in which several subjects were administered viral inoculum and gene expression time course data were collected over a period of several days. Using these data, we may infer relationships between genes and symptoms and examine how the human host response to viral infection evolves with time.

## Methods

### Mathematical constrained model

Let **y**_*i*_ represent a gene microarray vector of *G* gene expression levels. The elements of **y**_*i*_ have units of hybridization abundance levels with non-negative values. In the context of gene expression data, the starting point for Bayesian linear unmixing is the linear mixing model (LMM)

(3) $$y_{i}=\overset{R}{\underset{r=1}{\sum }}m_{r}a_{r,i}+n_{i},$$

where *R* is the number of distinct gene signatures that can be present in the chip, **m**_*r*_ = [*m*_1, *r*_, …, *m*_*G*, *r*_]^*T*^ is the *r*-th gene signature vector, *m*_*g*, *r*_ ≥ 0 is the strength of the *g*-th gene (*g* = 1, …, *G*) in the *r*-th signature (*r* = 1, …, *R*), and *a*_*r*, *i*_ is the relative contribution of the *r*-th signature vector to the *i*-th sample **y**_*i*_, where *a*_*r*,*i*_ ∈ [0, 1] and $\overset{R}{\underset{r=1}{\sum }}a_{r,i}=1$. Here **n**_*i*_ denotes the residual error of the LMM representation. For a matrix of *N* data samples $Y=\left[y_{1},\ldots ,y_{N}\right]\in R^{G\times N}$, the LMM can be rewritten with matrix notations

(4) Y=MA+N,

where $M=\left[m_{1},\ldots ,m_{R}\right]\in R^{G\times R}$, $A=\left[a_{1},\ldots ,a_{N}\right]\in R^{R\times N}$ and $N=\left[n_{1},\ldots ,n_{N}\right]\in R^{G\times N}$ represent the factor score matrix, the factor loading matrix and the noise matrix, respectively. The matrices **M**, **A** satisfy positivity and sum-to-one constraints defined by

(5) $$m_{g,r}\geq 0,\;\;\;a_{r,i}\geq 0,\;\;\;\text{and}\;\;\;[1,\ldots ,1]A=[1,\ldots ,1],$$

where *m*_*g*, *r*_ denotes the (*g*, *r*)-th element of matrix **M**. The constraints (5) arise naturally when dealing with positive data for which one is seeking the relative contribution of positive factors that have the same numerical characteristics as the data, i.e., the signature **m**_*r*_ is itself interpretable as a vector of hybridization abundances.

The objective of linear unmixing is to simultaneously estimate the factor matrix **M** and the factor score matrix **A** from the available *N* data samples. The representation (1) is rank deficient since **A** has rank *N* − 1. This presents algorithmic challenges for solving the unmixing problem. Several algorithms have been proposed in the context of hyperspectral imaging to solve similar problems [6,19]. Most of these algorithms perform unmixing in a two step procedure where **M** is estimated first using an *endmember extraction algorithm* (EEA) followed by a constrained linear least squares step to estimate **A**. A common (but restrictive) assumption in these algorithms is that some samples in the dataset are “pure” in the sense that the linear combination of (2) contains a unique factor, say **m**_*r*_, with factor score *a*_*r*, *i*_. Recently, this assumption has been relaxed by applying a hierarchical Bayesian approach, called Bayesian linear unmixing (BLU). The resulting algorithm requires the number *R* of factors to be specified (see [13] for details). Here we extend BLU to a fully unsupervised algorithm, called unsupervised BLU (uBLU), that estimates *R* using a birth-death model and a Gibbs sampler. The Gibbs sampler produces an estimate of the entire joint posterior distribution of the model parameters, resulting in a fully Bayesian estimator of the number of factors *R*, the factor loadings **M**, and the factor scores **A**. The uBLU model is described in the next subsection and the Gibbs sampling algorithm is given in the Appendix. In the Results and discussion section below we demonstrate the performance advantages of uBLU as a factor analysis model for simulated and real gene expression data.

### Unsupervised Bayesian linear unmixing algorithm

The BLU algorithm studied in [13] generates samples distributed according to the posterior distribution of **M** and **A** given the number *R* of factors for appropriate prior distributions assigned to the mixing parameters in (2). First, the residual errors **n**_*i*_ in (2) are assumed to be independent identically distributed (i.i.d.) according to zero-mean Gaussian distributions: $n_{i}∼N\left(0_{G},\sigma^{2}I_{G}\right)$ for *i* = 1, …, *N*, where **I**_*G*_ denotes the identity matrix of dimension *G* × *G*.

The number of factors *R* to be estimated by the proposed uBLU algorithm is assigned a discrete uniform prior distribution on [2, …, *R*_max_]

(6) $$P[R=k]=\frac{1}{R_{\text{max}}-1},\;\text{for}\;R=2,\ldots ,R_{\text{max}},$$

where *R*_max_ is the maximal number of factors present in the mixture.

Because of the constraints in (5), the data samples **y**_*i*_ (*i* = 1, …, *N*) live in a lower-dimensional subspace of $R^{K}$ (whose dimension is upper-bounded by *K* − 1) denoted as $V_{K-1}$ (*R*_max_ − 1 ≤ *K* ≤ *G*). This subspace can be identified by a standard dimension reduction procedure, such as PCA. Hence, instead of estimating the factor loadings $m_{r}\in R^{G}$ (*r* = 1, …, *R*), we propose to estimate their corresponding projections $t_{r}\in R^{K}$ onto this subspace. Specifically, these projections can be represented as

(7) $$t_{r}=P(m_{r}-\bar{y})$$

where $\bar{y}=\frac{1}{N}\overset{N}{\underset{i=1}{\sum }}y_{i}$ is the empirical mean of the data matrix **Y** and **P** is the (*K* − 1) × *G* appropriate projection matrix that projects onto $V_{K-1}$, which can be constructed from the principal eigenvectors of the empirical covariance matrix of **Y**. This dimension reduction procedure allows us to work in a lower-dimensional subspace without loss of information, and reduces significantly the computational complexity of the Bayes estimator of the factor loadings. A multivariate Gaussian distribution (MGD) truncated on a subset $T_{r}$ is chosen as prior distribution for the projected factors **t**_*r*_. The subset $T_{r}$ is defined in order to ensure the non-negativity constraint on **m**_*r*_ (see [13])

(8) $$t_{r}\in T_{r}\Leftrightarrow \{m_{g,r}\geq 0,\forall g=1,\ldots ,G\}.$$

More precisely, $T_{r}$ is obtained by noting that $m_{r}=P^{-1}t_{r}+\bar{y}$ and by looking for the vectors **t**_*r*_ such that all components of $P^{-1}t_{r}+\bar{y}$ are non-negative. To estimate the mean vectors **e**_*r*_ of these truncated MGDs, one can use a standard endmember extraction algorithm (EEA) common in hyperspectral imaging, e.g. N-FINDR [19]. To summarize, the prior distribution for the projected factor **t**_*r*_ is

(9) $$t_{r}|e_{r},s_{r}^{2}∼N_{T_{r}}\left(e_{r},s_{r}^{2}I_{R-1}\right)$$

where $N_{T_{r}}\left(e_{r},s_{r}^{2}I_{R-1}\right)$ denotes the truncated MGD with mean vector **e**_*r*_ and covariance matrix $s_{r}^{2}I_{R-1}$, with $s_{r}^{2}$ a fixed hyperparameter. Assuming the vectors **t**_*r*_, for *r* = 1, …, *R*, are *a priori* independent, the prior distribution for the projected factor matrix **T** = [**t**_1_,…,**t**_*R*_] is

(10) $$f\left(T|E,s^{2},R\right)\propto \overset{R}{\underset{r=1}{\prod }}\exp\left[-\frac{{∥t_{r}-e_{r}∥}^{2}}{2\overset{2}{\underset{r}{s}}}\right]1_{T_{r}}\left(t_{r}\right)$$

where ∝ stands for “proportional to”, ∥·∥ is the standard *l*_2_-norm, $1_{X}(\cdot )$ denotes the indicator function on the set X, **E** = [**e**_1_, …, **e**_*R*_] and $s^{2}=\left[s_{1}^{2},\ldots ,s_{R}^{2}\right]$.

The sum-to-one constraint for the factor scores **a**_*i*_, for each observed sample *i* (*i* = 1, …, *N*), allows this vector **a**_*i*_ to be rewritten as

(11) ai=a1:R−1,iaR,iwitha1:R−1,i=a1,i,…,aR−1,iT,

and $a_{R,i}=1-\overset{R-1}{\underset{r=1}{\sum }}a_{r,i}$. Note here that any component of **a**_*i*_ could be expressed as a function of the others, i.e., $a_{r,i}=1-\underset{k\neq r}{\sum }a_{k,i}$. The last component *a*_*R*, *i*_ has been chosen here for notation simplicity. To ensure the positivity constraint, the subvectors **a**_1:*R* − 1, *i*_ must belong to the simplex

(12) $$S=\{a_{1:R-1,i}\;|{∥a_{1:R-1,i}∥}_{1}\leq 1\;\text{and}\;a_{i}≽0\},$$

where ∥·∥_1_ is the *l*_1_ norm (${∥a_{i}∥}_{1}=\overset{R}{\underset{r=1}{\sum }}|a_{r,i}|$) and **a**_*i*_≽**0** stands for the set of inequalities {*a*_*r*,*i*_ ≥ 0}_*r* = 1, …, *R*_. Following the model in [13], we propose to assign uniform distributions over the simplex S as priors for the subvectors **a**_1:*R* − 1, *i*_ (*i* = 1, …, *N*), i.e.,

(13) $$f\left(a_{1:R-1,i}|R\right)=1_{S}\left(a_{1:R-1,i}\right).$$

For the prior distribution on the variance *σ*^2^ of the residual errors we chose a conjugate inverse-Gamma distribution with parameters *ν* / 2 and *γ* / 2

(14) $$\sigma^{2}|\nu ,\gamma ∼IG\left(\frac{\nu }{2},\frac{\gamma }{2}\right).$$

The shape parameter *ν* is a fixed hyperparameter whereas the scale parameter *γ* will be adjustable, as in [13]. A non-informative Jeffreys’ prior is chosen as prior distribution for the hyperparameter *γ*, i.e.,

(15) $$f\left(\gamma \right)\propto \frac{1}{\gamma }1_{R^{+}}(\gamma ).$$

The resulting hierarchical structure of the proposed uBLU model is summarized in the directed acyclic graph (DAG) presented in Additional file 1: Figure S1.

The model defined in (1) and the Gaussian assumption for the noise vectors **n**_1_, …, **n**_*N*_ allow the likelihood of **y**_1_, …, **y**_*N*_ to be determined

(16) $$f\left(Y|\Theta \right)={\left(\frac{1}{2\pi \sigma^{2}}\right)}^{\frac{\text{GN}}{2}}\exp\left[-\frac{\overset{N}{\underset{i=1}{\sum }}{∥y_{i}-{\mathrm{Ma}}_{i}∥}^{2}}{2\sigma^{2}}\right].$$

Multiplying this likelihood by the parameter priors defined in (10), (13), (14) and (6), and integrating out the nuisance parameter *γ*, the posterior distribution of the unknown parameter vector **Θ** = {**M**, **A**, *σ*^2^, *R*} can be expressed as

(17) $$\begin{array}{ll} \;f\left(\Theta |Y\right) & =\int f\left(\Theta ,\gamma |Y\right)\mathrm{d\gamma}\\ & \propto \int f\left(Y|\Theta \right)f\left(\Theta |\gamma \right)f\left(\gamma \right)\mathrm{d\gamma .} \end{array}$$

Considering the parameters to be *a priori* independent, the following result can be obtained

(18) $$f\left(\Theta |\gamma \right)=f\left(A|R\right)f\left(T|E,s^{2},R\right)f\left(\sigma^{2}|\nu ,\gamma \right)f\left(R\right)$$

where *f*(**A**|*R*), *f*(**T**|**E**, **s**^2^, *R*) and *f*(*σ*^2^|*ν*, *γ*) are respectively the prior distributions of the factor score matrix **A**, the projected factor matrix **T** and the noise variance *σ*^2^ previously defined.

Due to the constraints enforced on the data, the posterior distribution *f*(**M**, **A**, *R*|**Y**) obtained from the proposed hierarchical structure is too complex to derive analytical expressions of the Bayesian estimators, e.g., the minimum mean square (MMSE) and maximum a posteriori (MAP) estimators. In such case, it is natural to use Markov chain Monte Carlo (MCMC) methods [20] to generate samples **M**^(*t*)^, **A**^(*t*)^ and *R*^(*t*)^ asymptotically distributed according to *f*(**M**, **A**, *R*|**Y**). However, the dimensions of the factor loading matrix **M** and the factor score matrix **A** depend on the unknown number *R* of signatures to be identified. As a consequence, sampling from *f*(**M**, **A**, *R*|**Y**) requires exploring parameter spaces of different dimensions. To solve this dimension matching problem, we include a birth/death process within the MCMC procedure. Specifically, a birth, death or switch move is chosen at each iteration of the algorithm (see the Appendix and [21]). This birth-death model differs from the classical reversible-jump MCMC (RJ-MCMC) (as defined in [21]) in the sense that, for the birth-death model, each move is accepted or rejected at each iteration using the likelihood ratio between the current state and the new state proposed by the algorithm. The factor matrix **M**, the factor score matrix **A** and the noise variance *σ*^2^ are then updated, conditionally upon the number of factors *R*, using Gibbs moves.

After a sufficient number of iterations (N_mc_ iterations, including a burn-in period of N_bi_ iterations), the traditional Bayesian estimators (e.g., MMSE and MAP) can be approximated using the generated samples **M**^(*t*)^, **A**^(*t*)^ and *R*^(*t*)^. First, the generated samples are used to approximate the MAP estimator of the number of factors

(19) $$\begin{array}{ll} {\hat{R}}_{\text{MAP}} & =\underset{k\in \{2,\ldots ,R_{\text{max}}\}}{\mathrm{argmax}}\;P[R=k|Y]\\ & \approx \underset{k\in \{2,\ldots ,R_{\text{max}}\}}{\mathrm{argmax}}\;\frac{N_{k}}{N_{r}} \end{array}$$

where *N*_*k*_ is the number of generated samples $R^{\left(N_{\text{bi}}+1\right)},\ldots ,R^{\left(N_{\text{mc}}\right)}$ satisfying *R*^(*t*)^ = *k* and $N_{r}=N_{\text{mc}}-N_{\text{bi}}$. Then, conditioned on ${\hat{R}}_{\text{MAP}}$, the joint MAP estimator $\left({\hat{M}}_{\text{MAP}},{\hat{A}}_{\text{MAP}}\right)$ of the factor and factor score matrices is determined as follows

(20) $$\;\left({\hat{M}}_{\text{MAP}},{\hat{A}}_{\text{MAP}}\right)\;\approx \;\underset{t=N_{\text{bi}}+1,\ldots ,N_{\text{mc}}}{\mathrm{argmax}}\;\;f\;\left(M^{\left(t\right)},A^{\left(t\right)}|Y,R\;={\hat{R}}_{\text{MAP}}\;\right).$$

## Results and discussion

The proposed method consists of estimating simultaneously the matrices **M** and **A** defined in (1), under the positivity and sum-to-one constraints mentioned previously, in a fully unsupervised framework, i.e., the number of factors *R* is also estimated from the data. A Gibbs sampler algorithm is designed that generates samples distributed according to the posterior distribution associated to the proposed uBLU model. For more details about the Gibbs sampling strategy, see the Appendix.

### Simulations on synthetic data

To illustrate the performance of the proposed Bayesian factor decomposition, we first present simulations conducted on synthetic data. More extensive simulation results are reported in the Additional file 1.

#### Simulation scenario

Several synthetic datasets $D_{1},\ldots ,D_{4}$ were generated. The experiments presented here correspond to the expression values of *G* = 512 genes (for datasets $D_{1}$, $D_{3}$ and $D_{4}$) or *G*=12000 genes (for dataset $D_{2}$) with *N* = 128 samples. Each sample is composed of exactly *R*=3 factors mixed using the linear mixing model in (1). The factors of the first dataset $D_{1}$ have been generated so that only a few genes affect each factor. For the second dataset $D_{2}$, realistic factors have been extracted from real genetic datasets. The third dataset $D_{3}$ has been generated enforcing the factors to be orthogonal but not necessarily positive whereas in the forth dataset, $D_{4}$, factors are orthogonal and positive. These simulation conditions are summarized in Table 1.

**Table 1:** **Synthetic datasets**$D_{1},\ldots ,D_{4}$

	
_1_	Peaky factors
_2_	Realistic factors
_3_	Orthogonal factors
_4_	Orthogonal and positive factors

In each case, the *R* = 3 factors were mixed in random proportions (factor scores), with positivity and sum-to-one constraints. All synthetic datasets were corrupted by an i.i.d. Gaussian noise sequence. The signal-to-noise ratio is SNR_*i*_ = 20 dB where${\text{SNR}}_{i}=G^{-1}\sigma^{-2}{∥\overset{R}{\underset{r=1}{\sum }}m_{r}a_{r,i}∥}^{2}$ for each sample *i*(*i* = 1, …, *N*).

#### Proposed method (uBLU)

The first step of the algorithm consists of estimating the number of factors *R* involved in the mixture, and hence determining the dimensions of the matrices **M** and **A**, using the *maximum a posteriori* (MAP) estimator ${\hat{R}}_{\text{MAP}}$. The second step of the algorithm consists of estimating the unknown model parameters (**M**, **A** and *σ*^**2**^**) given**${\hat{R}}_{\text{MAP}}$. The estimated posterior distributions of the unknown model parameters are given in Additional file 1: Figure S5 and validate the proposed Bayesian model.

The burn-in period and number of Gibbs samples were determined using quantitative methods described in the Additional file 1: Section “Convergence diagnosis”.

#### Comparison to other methods

The performance of the proposed uBLU algorithm is compared with other existing factor decomposition methods including PCA, NMF, BFRM and GB-GMF by using the following criteria, which are common measures used to compare factor analysis algorithms, 

• the factor mean square errors (MSE)

$${\text{MSE}}_{r}^{2}=\frac{1}{G}{∥{\hat{m}}_{r}-m_{r}∥}^{2},\;r=1,\ldots ,R$$

where${\hat{m}}_{r}$ is the estimated *r*-th factor loading vector,

• the global MSE of factor scores

$${\text{GMSE}}_{r}^{2}=\frac{1}{N}\overset{N}{\underset{i=1}{\sum }}{\left({â}_{r,i}-a_{r,i}\right)}^{2},\;r=1,\ldots ,R$$

where ${â}_{r,i}$ is the estimated proportion of the *r*-th factor in the *i*-th sample,

• the reconstruction error (RE)

(21) $$\text{RE}=\frac{1}{\text{NG}}\overset{N}{\underset{i=1}{\sum }}{∥y_{i}-{\hat{y}}_{i}∥}^{2}$$

• where${\hat{y}}_{i}=\overset{R}{\underset{r=1}{\sum }}{\hat{m}}_{r}{â}_{r,i}$ is the estimate of **y**_*i*_, 

• the spectral angle distance (SAD) between **m**_*r*_ and its estimate${\hat{m}}_{r}$ for each factor *r* = 1, …, *R*

$${\text{SAD}}_{r}=\arccos\left(\frac{{\overset{ˆ}{m}}_{r}^{T}m_{r}}{∥{\overset{ˆ}{m}}_{r}∥∥m_{r}∥}\right)$$

where arccos(·) is the inverse cosine function,

• the global spectral angle distance (GSAD) between **y**_*i*_ (the *i*-th observation vector) and ${\hat{y}}_{i}$ (its estimate)

$$\text{GSAD}=\frac{1}{N}\overset{N}{\underset{i=1}{\sum }}\arccos\left(\frac{{\hat{y}}_{i}^{T}y_{i}}{∥{\hat{y}}_{i}∥∥y_{i}∥}\right),$$

• the computational time.

The proposed uBLU algorithm, the PCA, NMF and GB-GMF methods were implemented in Matlab 7.8.0 (R2009a). The BFRM software (version 2.0) was downloaded from [22] and implemented with default values for the parameters. All methods were implemented on an Intel(R) Core(TM)2 Duo processor.

Simulation results are reported in Tables 2, 3, 4 and 5. Note that the positivity and sum-to-one constraints that are enforced on the data for the proposed uBLU algorithm avoid the scale ambiguity inherent to any factor decomposition problem. Conversely, for the other factor decomposition methods (PCA, NMF, BFRM and GB-GMF), if {**M**, **A**} is an admissible solution, {**MB**, **B**^*T*^**A**} is also admissible for any scaling and permutation matrix **B**. Hence a re-scaling is required to identify appropriate permutations before computing MSEs and GMSEs. Moreover, when PCA, NMF, BFRM and GB-GMF methods are run for *R* = 4, we only considered the 3 factors yielding the 3 smallest SADs values.

**Table 2:** **Simulation results for dataset**$D_{1}$

**(a) *R *= 2**					
	**uBLU**	**PCA**	**NMF**	**BFRM**	**GB-GMF**
${\text{MSE}}_{r}^{2}(\times 10^{-2})$	**0.39**	N/A	N/A	205.99	267.42
	**0.60**	6.04	61.12	N/A	N/A
	**0.54**	0.97	9.78	325.58	67.14
${\text{GMSE}}_{r}^{2}(\times 10^{-3})$	**0.04**	N/A	N/A	64.39	226.58
	**0.04**	2.00	2.00	N/A	N/A
	**0.05**	0.30	0.28	75.87	41.33
${\text{SAD}}_{r}^{2}(\times 10^{-1})$	**0.46**	N/A	N/A	21.69	12.48
	**0.29**	3.49	3.50	N/A	N/A
	**0.28**	1.49	1.50	23.24	27.43
GSAD (×10^**−2**^)	**3.39**	20.38	20.38	24.04	37.35
RE	**0.18**	9.12	9.12	1.94	9.16
Time (*s*)	1.24×10^**3**^	**0.03**	0.71	47.15	0.39×10^**3**^
**(b) *R *= 3**					
	**uBLU**	**PCA**	**NMF**	**BFRM**	**GB-GMF**
${\text{MSE}}_{r}^{2}(\times 10^{-2})$	**0.39**	6.01	0.48	212.30	40.27
	0.60	6.53	**0.45**	681.42	147.74
	0.54	5.86	**0.28**	137.22	94.90
${\text{GMSE}}_{r}^{2}(\times 10^{-3})$	**0.04**	6.62	0.19	76.09	45.29
	0.04	2.40	**0.01**	142.72	17.37
	**0.05**	0.84	0.05	76.22	33.78
${\text{SAD}}_{r}^{2}(\times 10^{-1})$	**0.46**	1.86	0.53	10.68	11.86
	**0.29**	1.18	0.31	15.18	12.50
	0.28	1.36	**0.26**	5.33	13.96
GSAD (×10^**−2**^)	**3.37**	3.39	3.38	24.23	33.38
RE	**0.18**	**0.18**	0.18	1.84	0.18
Time (*s*)	1.24×10^**3**^	**0.10**	0.95	53.60	0.56×10^**3**^
**(c) *R *= 4**					
	**uBLU**	**PCA**	**NMF**	**BFRM**	**GB-GMF**
${\text{MSE}}_{r}^{2}(\times 10^{-2})$	**0.39**	6.02	87.78	205.66	195.89
	0.60	6.53	**0.45**	247.96	101.34
	0.54	8.03	**0.26**	330.01	68.69
${\text{GMSE}}_{r}^{2}(\times 10^{-3})$	**0.04**	23.82	26.56	64.59	57.58
	**0.04**	11.70	0.23	114.02	3.10
	**0.05**	6.37	18.04	75.47	27.72
${\text{SAD}}_{r}^{2}(\times 10^{-1})$	**0.46**	1.86	6.14	9.74	8.84
	**0.29**	1.18	0.31	22.15	26.80
	0.28	1.36	**0.26**	8.17	27.32
GSAD (×10^**−2**^)	3.39	**3.34**	3.36	28.62	29.23
RE	**0.18**	**0.18**	0.18	2.08	0.18
Time (*s*)	1.24×10^**3**^	**0.11**	0.96	63.88	0.70×10^**3**^

MSEs, GMSEs, SADs, GSADs, REs and computational times between the proposed uBLU algorithm and PCA, NMF, BFRM and GB-GMF methods.

**Table 3:** **Simulation results for dataset**$D_{2}$

**(a) *R *= 2**					
	**uBLU**	**PCA**	**NMF**	**BFRM**	**GB-GMF**
${\text{MSE}}_{r}^{2}$	**0.09**	1.97	N/A	N/A	N/A
	**0.14**	N/A	1.06	37.67	58.75
	0.14	**0.12**	26.68	52.09	150.09
${\text{GMSE}}_{r}^{2}(\times 10^{-1})$	0.34	**0.01**	N/A	N/A	N/A
	**0.15**	N/A	1.12	1.17	22.37
	**0.09**	0.94	6.24	0.62	1.18
${\text{SAD}}_{r}^{2}(\times 10^{-1})$	**0.39**	0.44	N/A	N/A	N/A
	**0.48**	N/A	1.32	16.53	13.34
	0.47	**0.44**	3.72	15.21	18.14
GSAD (×10^**−2**^)	1.51	**1.02**	1.53	37.99	129.40
RE (×10^**−2**^)	**0.64**	1.62	1.65	0.65	5.47
Time (*s*)	22.06×10^**3**^	**0.29**	32.02	4.07×10^**3**^	9.24×10^**3**^
**(b) *R *= 3**					
	**uBLU**	**PCA**	**NMF**	**BFRM**	**GB-GMF**
${\text{MSE}}_{r}^{2}$	**0.09**	1.97	14.87	24.41	61.00
	0.14	**0.01**	20.53	50.59	58.31
	0.14	**0.09**	14.02	35.89	65.11
${\text{GMSE}}_{r}^{2}(\times 10^{-1})$	0.34	**0.03**	0.34	1.41	4.80
	0.15	**0.02**	2.44	0.65	9.40
	0.09	**0.05**	0.92	1.19	5.40
${\text{SAD}}_{r}^{2}(\times 10^{-1})$	**0.39**	0.44	2.84	14.35	13.72
	0.48	**0.12**	4.75	15.47	13.62
	0.47	**0.37**	4.00	17.50	15.82
GSAD (×10^**−2**^)	**1.02**	1.02	1.49	29.29	129.29
RE (×10^**−2**^)	0.64	**0.63**	1.55	0.75	1.62
Time (*s*)	22.06×10^**3**^	**0.28**	45.91	5.37×10^**3**^	16.59×10^**3**^
**(c) *R *= 4**					
	**uBLU**	**PCA**	**NMF**	**BFRM**	**GB-GMF**
${\text{MSE}}_{r}^{2}$	**0.09**	1.97	13.13	24.25	64.90
	0.14	**0.01**	20.53	50.52	64.09
	0.14	**0.09**	14.02	28.32	69.99
${\text{GMSE}}_{r}^{2}(\times 10^{-1})$	0.34	**0.09**	0.20	1.42	15.12
	**0.15**	0.48	1.00	0.65	9.55
	0.09	**0.05**	0.44	1.31	7.73
${\text{SAD}}_{r}^{2}(\times 10^{-1})$	**0.39**	0.44	2.54	14.74	14.53
	0.48	**0.13**	5.52	15.45	14.55
	0.47	**0.37**	4.79	16.45	16.17
GSAD (×10^**−2**^)	1.02	**1.01**	1.06	40.36	129.29
RE (×10^**−2**^)	0.64	**0.63**	0.69	0.86	1.50
Time (*s*)	22.06×10^**3**^	**0.54**	55.86	5.59×10^**3**^	16.59×10^**3**^

MSEs, GMSEs, SADs, GSADs, REs and computational times between the proposed uBLU algorithm and PCA, NMF, BFRM and GB-GMF methods.

**Table 4:** **Simulation results for dataset**$D_{3}$

**(a) *R *= 2**					
	**uBLU**	**PCA**	**NMF**	**BFRM**	**GB-GMF**
${\text{MSE}}_{r}^{2}(\times 10^{-3})$	**0.01**	0.83	0.82	N/A	1.14
	0.85	**0.80**	0.92	1.34	2.30
	**1.15**	N/A	N/A	1.36	N/A
${\text{GMSE}}_{r}^{2}(\times 10^{-2})$	7.75	**7.29**	7.72	N/A	8.94
	7.76	**0.47**	0.48	12.30	11.86
	**9.84**	N/A	N/A	11.05	N/A
${\text{SAD}}_{r}^{2}(\times 10^{-1})$	**0.59**	7.09	7.04	N/A	15.55
	7.13	**6.71**	7.19	8.41	16.43
	8.71	N/A	N/A	**8.54**	N/A
GSAD (×10^**−1**^)	3.23	**2.58**	2.59	6.59	15.26
RE (×10^**−4**^)	3.11	0.70	0.70	**0.47**	2.50
Time (*s*)	1.59×10^**3**^	**0.01**	0.70	42.02	0.40×10^**3**^
**(b) *R *= 3**					
	**uBLU**	**PCA**	**NMF**	**BFRM**	**GB-GMF**
${\text{MSE}}_{r}^{2}(\times 10^{-3})$	**0.01**	0.15	0.15	1.74	1.20
	0.85	1.02	**0.76**	1.76	2.26
	1.15	1.57	**1.03**	1.55	2.40
${\text{GMSE}}_{r}^{2}(\times 10^{-2})$	7.75	14.89	**2.80**	11.40	14.09
	7.76	**0.11**	0.40	12.11	12.33
	9.84	**0.11**	0.30	10.94	12.76
${\text{SAD}}_{r}^{2}(\times 10^{-1})$	**0.59**	2.60	2.47	11.34	15.76
	7.13	7.16	**6.59**	9.45	16.40
	8.71	8.80	**7.67**	9.06	15.66
GSAD (×10^**−1**^)	3.23	**2.58**	1.71	6.88	15.20
RE (×10^**−4**^)	3.11	**0.27**	0.29	0.49	2.44
Time (*s*)	1.59×10^**3**^	**0.10**	1.24	59.72	0.54×10^**3**^
**(c) *R *= 4**					
	**uBLU**	**PCA**	**NMF**	**BFRM**	**GB-GMF**
${\text{MSE}}_{r}^{2}(\times 10^{-3})$	**0.01**	0.02	1.43	1.43	1.19
	**0.85**	1.48	5.49	3.92	2.06
	1.15	1.68	**0.90**	1.88	2.33
${\text{GMSE}}_{r}^{2}(\times 10^{-2})$	**7.75**	13.78	20.56	16.66	13.15
	7.76	**4.35**	12.36	15.34	11.75
	9.84	3.99	**2.67**	11.25	13.29
${\text{SAD}}_{r}^{2}(\times 10^{-1})$	**0.59**	0.97	10.27	10.24	15.97
	**7.13**	7.93	15.78	16.45	14.92
	8.71	8.66	**6.93**	10.98	15.89
GSAD (×10^**−1**^)	3.23	**1.17**	1.20	5.51	15.98
RE (×10^**−4**^)	3.11	**0.16**	0.16	0.41	2.45
Time (*s*)	1.59×10^**3**^	**0.13**	1.15	67.71	0.69×10^**3**^

MSEs, GMSEs, SADs, GSADs, REs and computational times between the proposed uBLU algorithm and PCA, NMF, BFRM and GB-GMF methods.

**Table 5:** **Simulation results for dataset**$D_{4}$

**(a) *R *= 2**					
	**uBLU**	**PCA**	**NMF**	**BFRM**	**GB-GMF**
${\text{MSE}}_{r}^{2}(\times 10^{-2})$	**0.02**	N/A	5.12	N/A	N/A
	1.61	**0.01**	3.59	15.35	18.69
	**0.05**	0.44	N/A	14.42	19.20
${\text{GMSE}}_{r}^{2}(\times 10^{-1})$	**0.28**	N/A	3.23	N/A	N/A
	0.87	**0.02**	2.65	0.33	1.62
	0.69	0.76	N/A	**0.50**	1.30
${\text{SAD}}_{r}^{2}(\times 10^{-1})$	**0.34**	N/A	4.25	N/A	N/A
	3.08	**0.17**	3.71	14.90	14.89
	**0.51**	0.68	N/A	15.59	15.70
GSAD (×10^**−2**^)	**4.97**	5.24	5.25	157.09	156.19
RE (×10^**−4**^)	**4.49**	4.88	4.89	19.34	8.48
Time (*s*)	1.61×10^**3**^	**0.02**	1.36	35.29	0.40×10^**3**^
**(b) *R *= 3**					
	**uBLU**	**PCA**	**NMF**	**BFRM**	**GB-GMF**
${\text{MSE}}_{r}^{2}(\times 10^{-2})$	0.02	**0.01**	6.18	18.38	21.63
	1.61	**0.01**	4.79	16.10	19.55
	**0.05**	0.09	4.21	15.04	19.85
${\text{GMSE}}_{r}^{2}(\times 10^{-1})$	0.28	**0.05**	1.67	1.44	1.29
	0.87	**0.05**	1.01	0.37	1.75
	0.69	**0.05**	0.94	0.26	1.17
${\text{SAD}}_{r}^{2}(\times 10^{-1})$	0.34	**0.27**	4.12	15.21	15.65
	3.08	**0.17**	4.09	15.26	15.90
	0.51	**0.32**	4.16	16.07	15.36
GSAD (×10^**−2**^)	4.97	**4.95**	4.99	157.08	154.80
RE (×10^**−4**^)	4.49	**4.34**	4.36	25.00	8.48
Time (*s*)	1.61×10^**3**^	**0.10**	1.78	41.05	0.55×10^**3**^
**(c) *R *= 4**					
	**uBLU**	**PCA**	**NMF**	**BFRM**	**GB-GMF**
${\text{MSE}}_{r}^{2}(\times 10^{-2})$	0.02	**0.01**	6.98	17.51	21.60
	1.61	**0.01**	7.30	15.07	19.03
	**0.05**	0.07	4.27	14.55	19.14
${\text{GMSE}}_{r}^{2}(\times 10^{-1})$	0.28	**0.22**	0.65	0.75	1.29
	0.87	**0.51**	0.91	0.77	1.18
	0.69	**0.05**	0.56	0.56	1.33
${\text{SAD}}_{r}^{2}(\times 10^{-1})$	0.34	**0.27**	4.41	15.61	15.51
	3.08	**0.19**	4.81	16.31	14.77
	0.51	**0.33**	4.00	15.84	15.26
GSAD (×10^**−2**^)	4.97	**4.91**	4.94	156.76	162.63
RE (×10^**−4**^)	4.49	**4.30**	4.33	13.48	8.29
Time (*s*)	1.61×10^**3**^	**0.16**	1.56	48.22	0.70×10^**3**^

MSEs, GMSEs, SADs, GSADs, REs and computational times between the proposed uBLU algorithm and PCA, NMF, BFRM and GB-GMF methods.

These results show that the uBLU method is more flexible since it provides better unmixing performance across all of the considered synthetic datasets $D_{1},\ldots ,D_{4}$ as compared to other existing factorization methods (PCA, NMF, BFRM and GB-GMF). Moreover, uBLU has the following advantages: i) it is fully unsupervised and does not require the number of factors to be specified as a prior knowledge, ii) due to the constraints, the factors and factor scores are estimated without scale ambiguity. The disadvantage is the execution time: uBLU requires more computation due to the Gibbs sampling.

### Evaluation on gene expression data

Here the proposed algorithm is illustrated on a real time-evolving gene expression data from recent viral challenge studies on influenza A/H3N2/Wisconsin. The data are available at GEO, accession number GSE30550.

#### Details on data collection

**Figure 1:**
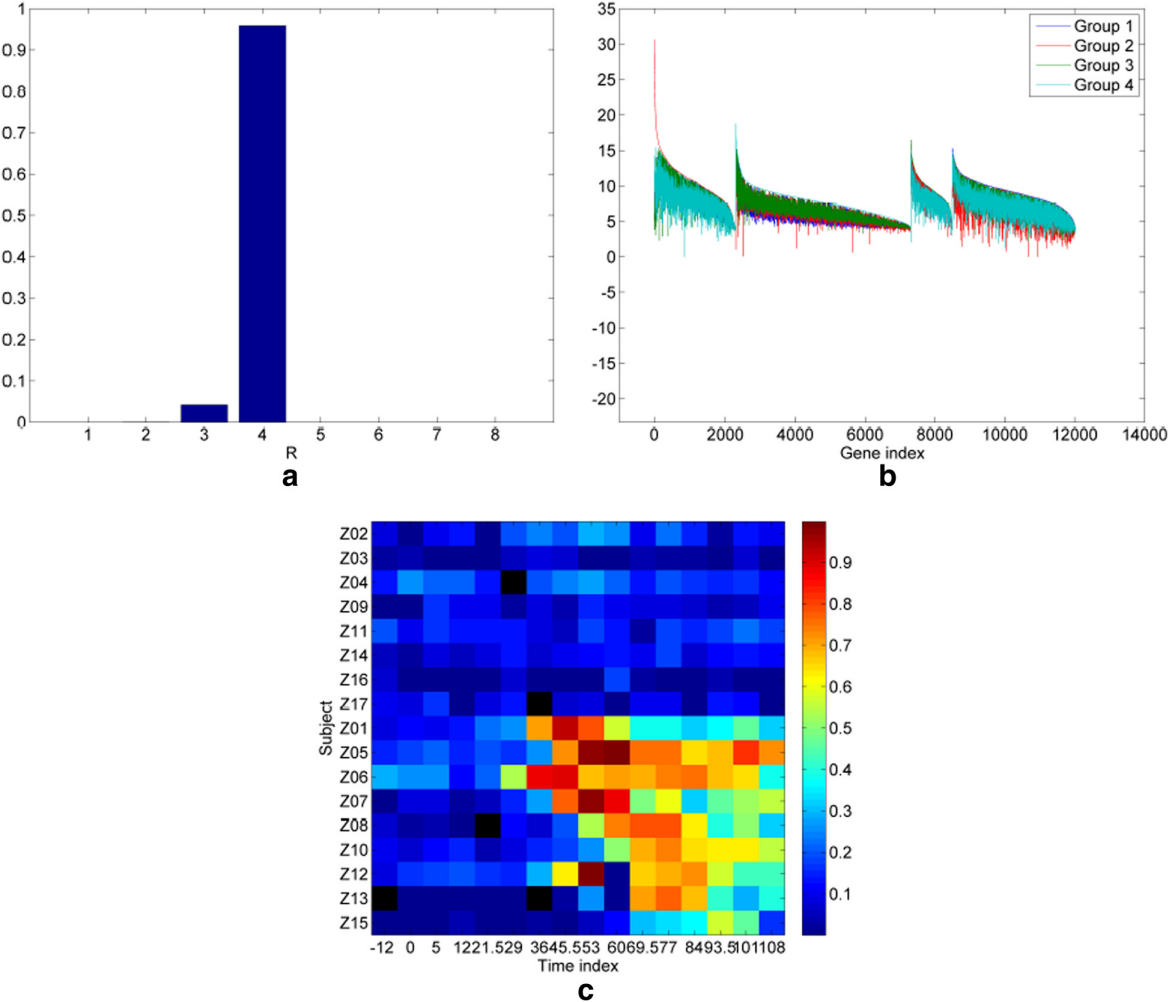
**Experimental results on the H3N2 viral challenge dataset of gene expression profiles.** (**a**) Estimated posterior distribution of the number of factors *R*. (**b**) Factor loadings ranked by decreasing dominance. (**c**) Heatmap of the factor scores of the inflammatory component clearly separates symptomatic subjects (bottom 9 rows) and the time course of their molecular inflammatory response. The five black colored pixels indicate samples that were not assayed.

We briefly describe the dataset. For more details the reader is referred to [14,18]. H3N2 dataset consists of the gene expression levels of *N* = 267 Affymetrix chips collected on 17 healthy human volunteers experimentally infected with influenza A/Wisconsin/67/2005 (H3N2). A clinical symptom score was assigned to each sample by clinicians who participated in the study. Nine of the 17 subjects (those labeled Z01, Z05, Z06, Z07, Z08, Z10, Z12, Z13, and Z15 in Figure 1c) became clinically ill during the study. These labels are only used as ground truth to quantify performance and are not available to the uBLU algorithm. The challenge consists of inoculating intranasally a dose of 10^6^ TCID_50_ Influenza A manufactured and processed under current good manufacturing practices (cGMP) by Baxter BioScience. Peripheral blood microarray analysis was performed at multiple time instants corresponding to baseline (24 hours prior to inoculation with virus), then at 8 hour intervals for the initial 120 hours and then 24 hours for two further days. Each sample consisted of over *G* = 12000 gene expression values after standard microarray data normalization with RMA using the custom brain array cdf [14]. No other preprocessing was applied prior to running the five unsupervised methods (uBLU, PCA, NMF, BFRM, and GB-GMF).

#### Application of the proposed uBLU algorithm

The uBLU algorithm was run with N_mc_ = 10000 Monte Carlo iterations, including a burn-in period of N_bi_ = 1000 iterations. uBLU allows the posterior distribution of the number of factors *R*, depicted in Figure 1a, to be estimated. The results show that the MAP estimate of the number of factors is${\hat{R}}_{\text{MAP}}=4$ (more than 90% of the generated Gibbs samples of the number of factors were equal to 4).

**Figure 2:**
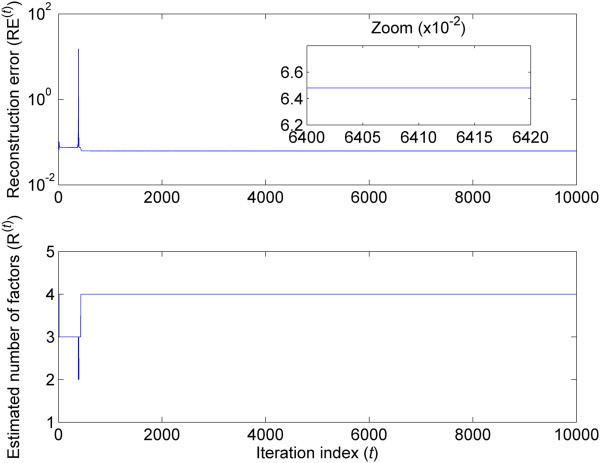
**Reconstruction error and estimated number of factors as a function of the number of iterations (H3N2 challenge data).** Top: Reconstruction error (RE^(*t*)^) computed from the observation matrix **Y** and the estimated matrices **M**^(*t*)^ and **A**^(*t*)^ as a function of the iteration index *t*. Bottom: Estimated number of factors *R*^(*t*)^ as a function of the iteration number *t*.

Figure 2 shows the reconstruction error RE^(*t*)^ as a function of the number of iterations (*t* = 1, …). The reconstruction errors are computed from the observed gene expression data matrix and the estimates of the factor and factor score matrix **M** and **A** at each iteration. Figure 2 also indicates that the number of burn-in and Monte Carlo samples N_bi_ = 1000 and N_mc_ = 10000 are sufficient.

The different factors are depicted in Figure 1b where the *G* genes have been reordered so that the dominant genes are grouped together in each factor. Factors are then ordered with respect to their maximum loading. Specifically, the *k*-th sharp peak in the figure occurs at the gene index that has maximal loading in factor *k*. Genes to the right of this dominant gene up to the (*k* + 1)-st peak also dominate in this *k*-th factor, but at a lower degree. uBLU identifies a strong factor (the first factor, in red) by virtue of its significantly larger proportion of highly dominant genes. Many of the genes in this strong factor are recognizable as immune response genes that regulate pattern recognition, interferon, and inflammation pathways in respiratory viral response. A very similar factor was found in a different analysis [14,18] of this dataset and here we call it the “*inflammatory component*”.

The factor scores corresponding to this inflammatory component are shown in Figure 1c, where they are rendered as an image whose columns (respectively rows) index the subjects (respectively the different time sampling instants). Figure 1c shows that uBLU clearly separates the samples of subjects exhibiting symptoms (associated with the last 9 rows) from those who remain asymptomatic (associated with the first 8 rows), when the estimated number of factors is$\hat{R}=4$. Moreover, this symptom factor can be used to segment the data matrix into 3 states: pre-onset-symptomatic (before significant symptoms occur), post-onset-symptomatic and asymptomatic.

Furthermore, this inflammatory factor identified by the proposed uBLU algorithm is most highly represented in those samples associated with acute flu symptoms, as measured by modified Jackson scores (see [14], Figure 1B). The dominant gene contributors to this inflammatory component correspond to well-known transcription factors controlling immune response, inflammatory response and antigen presentation – see Table 6. The reader is referred to [14,18] for more details on clinical determination of symptom scores and biological significance of the inflammatory component genes.

**Table 6:** NCI-curated pathway associations of group of genes contributing to uBLU inflammatory component

**Pathway name**	**Genes**	**P-value**
IFN-gamma pathway	CASP1, CEBPB, IL1B, IRF1, IRF9, PRKCD, SOCS1, STAT1, STAT3	1.34e-09
PDGFR-beta signaling pathway	DOCK4, EIF2AK2, FYN, HCK, LYN, PRKCD, SLA, SRC, STAT1, STAT3, STAT5A, STAT5B	3.26e-08
IL23-mediated signaling events	CCL2, CXCL1, CXCL9, IL1B, STAT1, STAT3, STAT5A	2.18e-07
Signaling events mediated by TCPTP	EIF2AK2, SRC, STAT1, STAT3, STAT5A, STAT5B, STAT6	6.38e-07
Signaling events mediated by PTP1B	FYN, HCK, LYN, SRC, STAT3, STAT5A, STAT5B	2.40e-06
GMCSF-mediated signaling events	CCL2, LYN, STAT1, STAT3, STAT5A, STAT5B	3.70e-06
IL12-mediated signaling events	HLA-A, IL1B, SOCS1, STAT1, STAT3, STAT5A, STAT6	1.32e-05
IL6-mediated signaling events	CEBPB, HCK, IRF1, PRKCD, STAT1, STAT3	1.80e-05

NCI-curated pathway associations of group of genes contributing to uBLU inflammatory component, whose factor scores are shown in Figure 1 (Source: NCI pathway interaction database http://pid.nci.nih.gov). Genes in uBLU factor are significantly better represented in the NCI-curated pathways than the genes in NMF (compare p-values here to those in Table 8).

For comparison we applied a supervised version of the proposed uBLU algorithm to the H3N2 dataset. This was implemented by setting the number of factors to *R* = 4 and using the algorithm [13] to jointly estimate **M** and **A**. The inflammatory component found by the supervised algorithm was virtually identical to the one found by the proposed algorithm (uBLU) that automatically selects *R* = 4.

#### Comparison to other methods

The uBLU algorithm is compared with four matrix factorization algorithms, i.e. PCA, NMF, BFRM and GB-GMF methods.

**Figure 3:**
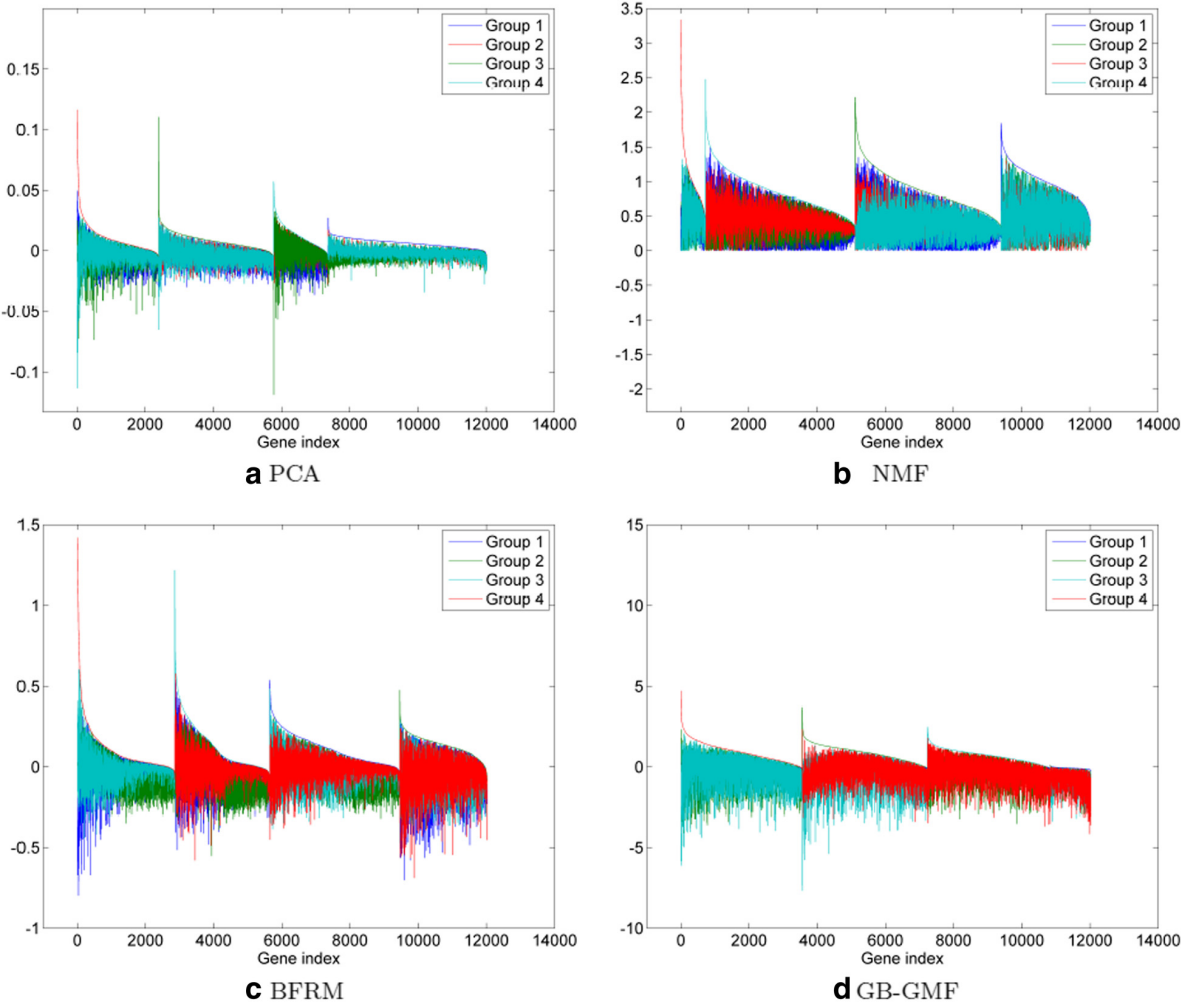
**Factor loadings ranked by decreasing dominance for H3N2 challenge data.** uBLU shows a particularly strong component (Figure 1b), the group *♯*1, that corresponds to the well-known inflammatory pathway. NMF and PCA algorithms also reveal an inflammatory component, but it includes fewer relevant genes than uBLU. See Figure 4 for the corresponding factor scores.

Figure 3 depicts the different factors, ordered so that the inflammatory group is the leftmost one (in red). The factor loadings obtained with NMF or PCA reveal the inflammatory component. However, there are fewer highly dominant genes in the NMF and PCA loadings for this factor as compared to uBLU. The BFRM and GB-GMF methods found four pathways, several overlapping with those of uBLU, NMF and PCA.

**Figure 4:**
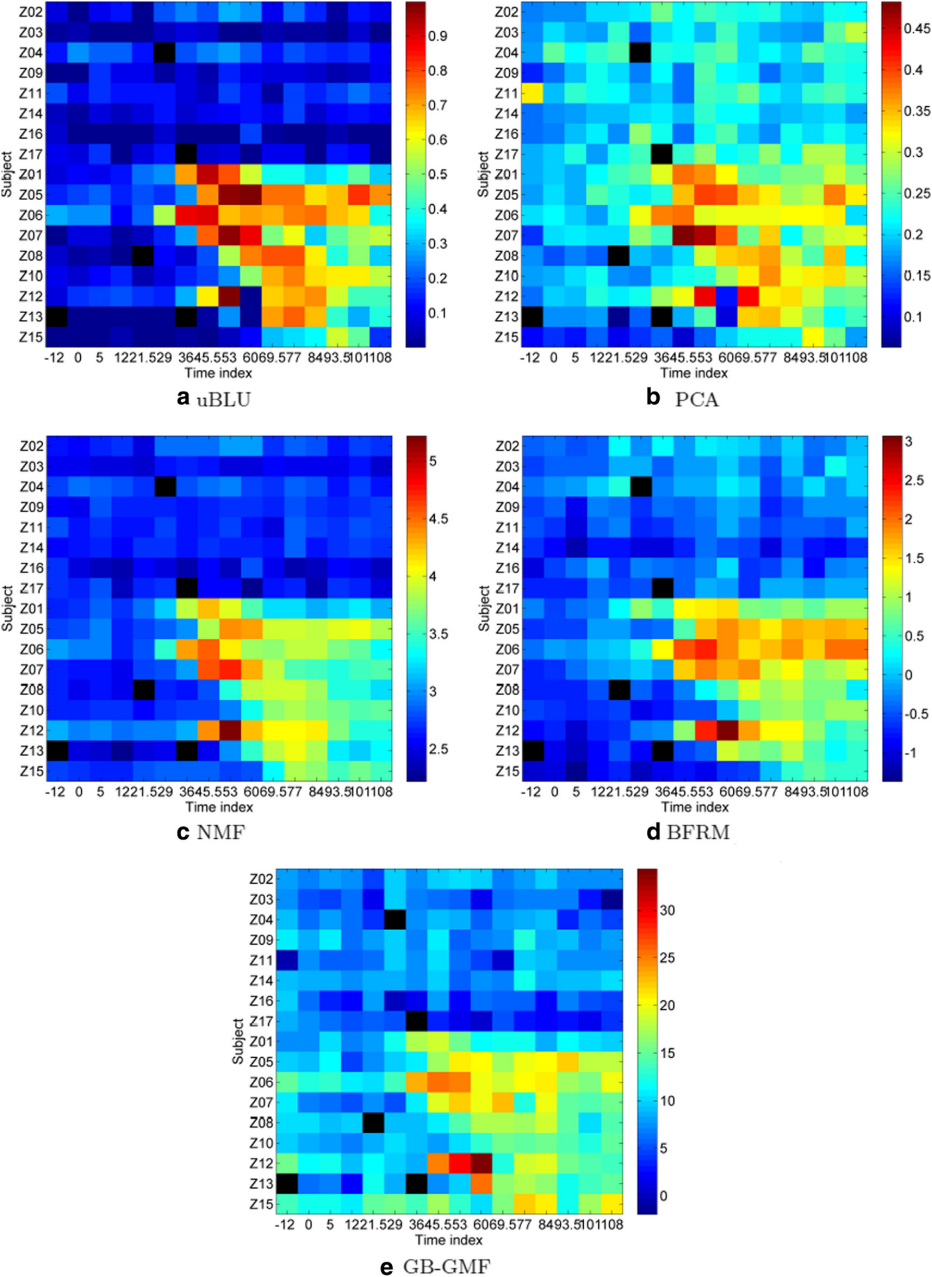
**Heatmaps of the factor scores of the inflammatory component for H3N2 challenge data.** The inflammatory factor determined by the proposed uBLU method (**a**) shows higher contrast between symptomatic and asymptomatic subjects than the other methods. The five black colored pixels of the heatmaps indicate samples that were not assayed.

The factor scores of the five matrix factorization methods corresponding to the inflammatory component are depicted in Figure 4. This figure shows that the uBLU and the NMF methods are better able to attain a high contrast separation between the acutely symptomatic samples and the other samples. This is confirmed by the evaluation of the Fisher criteria (22) between these two regions (see Table 7). Indeed, denote by (*μ*_pos_, *σ*^2^pos) the empirical mean and variance of the scores associated with the *N*_pos_ samples in the acute symptomatic state (bright colored samples in the lower right rectangle of Figure 1c). Denote by $\left(\mu_{\bar{\text{pos}}},{\sigma^{2}}_{\bar{\text{pos}}}\right)$ the same parameters for the remaining samples. The Fisher linear discriminant measure ([23], p. 119) is defined as

(22) $$\frac{{\left(\mu_{\text{pos}}-\mu_{\bar{\text{pos}}}\right)}^{2}}{N_{\text{pos}}{\sigma^{2}}_{\text{pos}}+(N-N_{\text{pos}}){\sigma^{2}}_{\bar{\text{pos}}}}.$$

**Table 7:** Simulation results for real H3N2 dataset

	**uBLU**	**PCA**	**NMF**	**BFRM**	**GB-GMF**
Fisher criteria (× 10^−2^) (22)	**6.20**	2.03	6.17	4.68	2.30
RE	**6.48.10**^**−2**^	4.89	7.31.10^−2^	4.82	9.51.10^−2^
Time	≈ 12 *h*	**1.5*s***	116 *s*	≈ 47 *min*	≈ 10 *h*
Number of iterations	10 000	N/A	5 000	10 000	500

Measure of the Fisher linear discriminant measure ([23], p. 119) between post-onset symptomatic samples and the other samples on heatmaps (Figure 4), reconstruction error (RE) between the observed data and the MAP estimators, computational times (for an implementation in MATLAB 7.8.0 (R2009a) on a 3 GHz Intel(R) Core(TM)2 Duo processor), and corresponding number of iterations.

To compare the biological relevance of the inflammatory genes found by uBLU to those found by the other methods we performed gene enrichment analysis (GEA). Here we only report GEA comparisons between uBLU and NMF. Tables 6 and 8 show the pathway enrichment associated with the top 200 genes found by uBLU and NMF, respectively, using NCI pathway interaction database (http://pid.nci.nih.gov). The uBLU genes are significantly better associated with the NCI-curated pathways than the NMF genes. In particular, the two most enriched pathways, IFN-gamma and PDGFR beta signaling, associated with the uBLU genes have much higher statistical significance (lower p-value) than any of the pathways associated with NMF.

**Table 8:** NCI-curated pathway associations of group of genes contributing to NMF inflammatory component

**Pathway name**	**Genes**	**P-value**
IL23-mediated signaling events	CCL2, CXCL1, CXCL9, IL1B, JAK2, STAT1, STAT5A	2.18e-07
IL12-mediated signaling events	GADD45B, IL1B, JAK2, MAP2K6, SOCS1, STAT1, STAT5A, STAT6	1.10e-06
IFN-gamma pathway	CASP1, IL1B, IRF9, JAK2, SOCS1, STAT1	1.07e-05
Signaling events mediated by TCPTP	EIF2AK2, PIK3R2, STAT1, STAT5A, STAT5B, STAT6	1.07e-05
IL27-mediated signaling events	IL1B, JAK2, STAT1, STAT2, STAT5A	1.22e-05
CXCR3-mediated signaling events	CXCL10, CXCL11, CXCL13, CXCL9, MAP2K6, PIK3R2	1.23e-05
GMCSF-mediated signaling events	CCL2, JAK2, STAT1, STAT5A, STAT5B	6.24e-05
PDGFR-beta signaling pathway	EIF2AK2, JAK2, PIK3R2, ARAP1, DOCK4, STAT1, STAT5A, STAT5B	1.38e-04

NCI-curated pathway associations of group of genes contributing to NMF inflammatory component, whose factor scores are shown in Figure 4 (Source: NCI pathway interaction database http://pid.nci.nih.gov). Genes in uBLU factor are significantly better represented in the NCI-curated pathways than the genes in NMF (compare p-values here to those in Table 6).

**Figure 5:**
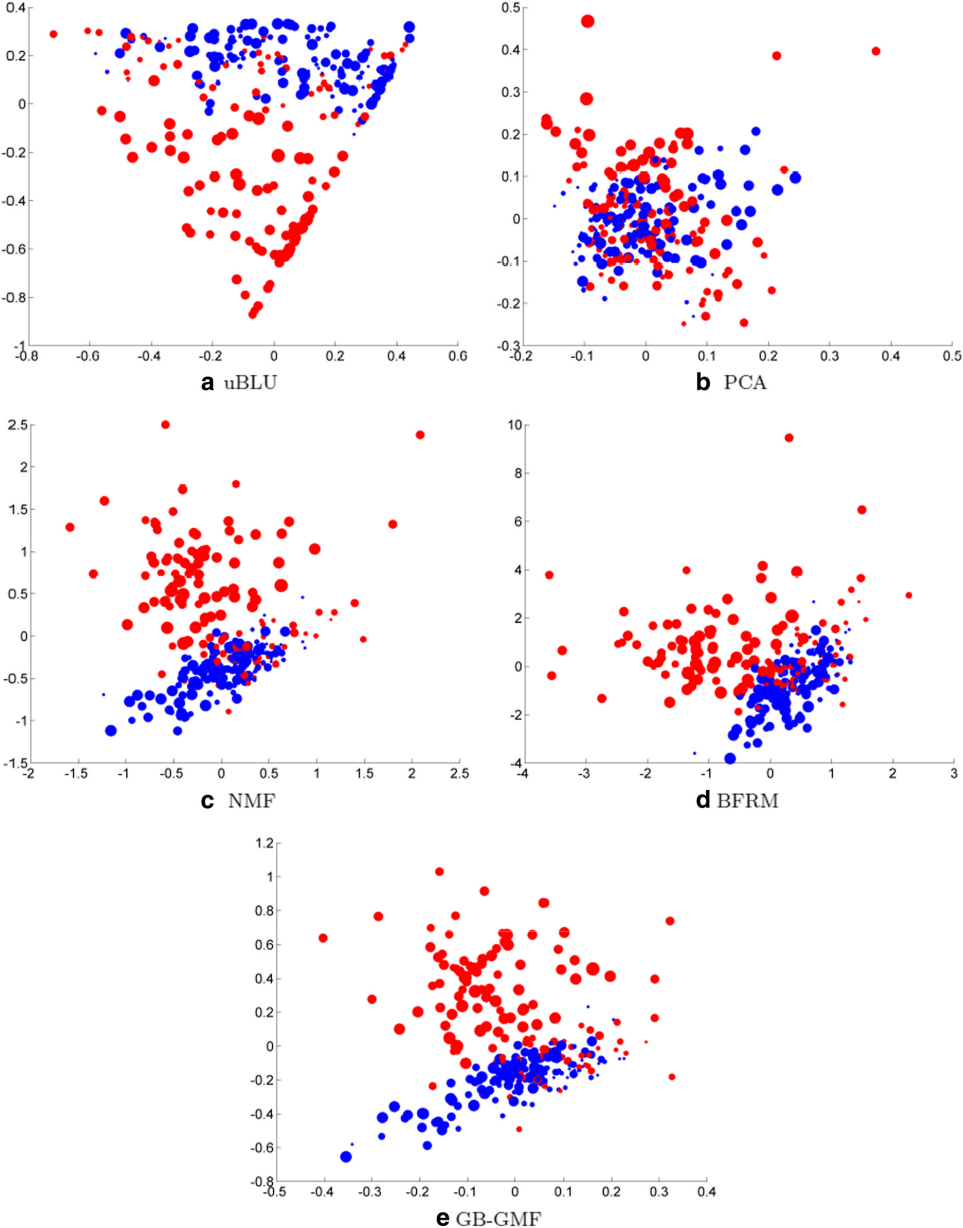
**Chip clouds after demixing for H3N2 challenge data.** These figures show the scatter of the four dimensional factor score vectors (projected onto the plane using MDS) for each algorithm that was compared to uBLU. uBLU, NMF and BFRM obtain a clean separation of samples of symptomatic (red points) and asymptomatic (blue points) subjects whereas the separation is less clear with PCA. In these scatter plots the size of a point is proportional to the time at which the sample was taken during challenge study.

Figure 5 shows how the factor scores of the dominant factor can be used as features to cluster samples. Euclidean multidimensional scaling (MDS) [24] is used to map the factor score vector for each sample as a coordinate in the plane. Each sample is embedded with a color and a size, denoting the state of the subject (asymptomatic subjects in blue, symptomatic subjects in red) and the time stamp, respectively. These figures show that uBLU can separate sick and healthy subjects, as well as or better than NMF, BFRM and GB-GMF.

One can conclude from these comparisons that, when applied on the H3N2 dataset, the proposed uBLU algorithm outperforms PCA, NMF, BFRM, and GB-GMF algorithms in terms of finding genes with higher pathway enrichment and achieving higher contrast of the acute symptom states.

The computational times required by the five considered matrix factorization methods, including the proposed uBLU algorithm, when applied to this real dataset, are reported in Table 7. The GB-GMF algorithm is slightly faster than the proposed algorithm but does not identify the inflammatory component or achieve good contrast of the acute symptom states in the H3N2 challenge study.

## Conclusions

This paper proposes a new Bayesian unmixing algorithm for discovering signatures in high dimensional biological data, and specifically for gene expression microarrays. An interesting property of the proposed algorithm is that it provides positive factor loadings to ensure positivity as well as sum-to-one constraints for the factor scores. The advantages of these constraints are that they lead to better discrimination between sick and healthy individuals, and they recover the inflammatory genes in a unique factor, the inflammatory component. The proposed algorithm is fully unsupervised in the sense that it does not depend on any labeling of the samples and that it can infer the number of factors directly from the observation data matrix. Finally, as any Bayesian algorithm, the Monte Carlo-based procedure investigated in this study provides point estimates as well as confidence intervals for the unknown parameters, contrary to many existing factor decomposition methods such as PCA or NMF.

Simulation results performed on synthetic and real data demonstrated significant improvements. Indeed, when applied to real time-evolving gene expression datasets, the uBLU algorithm revealed an inflammatory factor with higher contrast between subjects who would become symptomatic from those who would remain asymptomatic (as determined by comparing to ground truth clinical labels).

In this study, the time samples were modeled as independent. Future works include extensions of the proposed model to account for time dependency between samples.

## Appendix A: Gibbs sampler

This appendix provides more details about the Gibbs sampler strategy to generate samples$\left\{M^{\left(t\right)},A^{\left(t\right)},\sigma^{2^{\left(t\right)}},R^{\left(t\right)}\right\}$ distributed according to the joint distribution$f\left(M,A,\sigma^{2},R|Y\right)$ (the reader is referred to [25] for more details about the Gibbs sampler and MCMC methods). This joint distribution can be obtained by integrating out the hyperparameter *γ* from *f*(**Θ**, *γ*|**Y**) defined in (18) and can be written

(23) $$\begin{array}{ll} \;f\left(M,A,\sigma^{2},R|Y\right) & \propto f\left(Y|M,A,\sigma^{2},R\right)\\ & \times f\left(T|E,s^{2},R\right)\\ & \times f\left(A|R\right)\\ & \times f\left(\sigma^{2}\right)f\left(R\right) \end{array}$$

where the dimensions of the matrices **M**, **T**, and **A** depend on the unknown number of factors *R* and the priors have been defined in the Section “Methods”.

The different steps of the Gibbs sampler are detailed below.

### Inference of the number of factors

The proposed unsupervised algorithm includes a birth/death process for inferring the number of factors *R*, i.e., it generates samples *R* in addition to **M** and **A**. More precisely, at iteration *t* of the algorithm, a birth, death or switch move is randomly chosen with probabilities $b_{R^{\left(t\right)}}$, $d_{R^{\left(t\right)}}$ and $s_{R^{\left(t\right)}}$. The *birth* and *death* moves consist of increasing or decreasing by 1 the number *R* of factors using a reversible jump step (see [21] for more details), whereas the *switch* move does not change the dimension of *R* and requires the use of a Metropolis-Hastings acceptance procedure. Let consider a move, at iteration index *t*, from the state {**M**^(*t*)^, **A**^(*t*)^, *R*^(*t*)^} to the new state {**M**^**⋆**^, **A**^**⋆**^, *R*^**⋆**^}. The birth, death and switch moves are defined as follows, similar to those used in [26] (Algorithms 3, 4 and 5). 

• ***Birth *move:** When a birth move is proposed, a new signature **m**^**⋆**^ is randomly generated to build **M**^**⋆**^ = [**M**^(*t*)^, **m**^**⋆**^]. The new corresponding space is checked so that the signatures are sufficiently distinct and separate from one another. Then, a new factor score coefficient is drawn, for each vector **a**_*i*_ (*i* = 1, …, *N*), from a Beta distribution $ℬ\left(1,R^{\left(t\right)}\right)$, and the new factor score matrix, denoted as **A**^**⋆**^, is re-scaled to sum to one.

• ***Death *move:** When a death move is proposed, one of the factors of **M**^(*t*)^, and its corresponding factor score coefficients, are randomly removed. The remaining factor scores are re-scaled to ensure the sum-to-one constraint.

• ***Switch *move:** When a switch move is proposed, a signature **m**^**⋆**^ is randomly chosen and replaced with another signature randomly generated. If the new signature is too close to another, its corresponding factor scores are proportionately distributed among its closest factors. Indeed, the switch move consists of creating a new signature (birth move) and deleting another one (death move) in a faster single step.

Each move is then accepted or rejected according to an empirical acceptance probability: the likelihood ratio between the actual state and the proposed new state. The factor matrix **M**, the factor score matrix **A** and the noise variance *σ*^2^ are then updated, conditionally upon the number of factors *R*, using the following Gibbs steps.

### Generation of samples according to *f*(T|A, *σ*^**2**^, *R*, Y)

Sampling from the joint conditional *f*(**T**|**A**, *σ*^2^, *R*,**Y**) is achieved by updating each column of **T** using Gibbs moves. Let denote **T**_∖*r*_ the matrix **T** whose *r*-th column has been removed. The posterior distribution of **t**_*r*_ is the following truncated multivariate Gaussian distribution (MGD)

(24) $$t_{r}|T_{\setminus r},a_{r},\sigma^{2},Y∼N_{T_{r}}\left(\tau_{r},\Gamma_{r}\right)$$

where

(25) $$\begin{array}{ll} \;\Gamma_{r} & ={\left[\overset{N}{\underset{i=1}{\sum }}a_{r,i}^{2}P\Sigma^{-1}P^{T}+\frac{1}{s_{r}^{2}}{\text{I}}_{R}\right]}^{-1},\\ \;\tau_{r} & =\Gamma_{r}\left[\overset{N}{\underset{i=1}{\sum }}a_{r,i}P\Sigma^{-1}\epsilon_{r,i}+\frac{1}{s_{r}^{2}}e_{r}\right],\\ \;\epsilon_{r,i} & =y_{i}-a_{r,i}\bar{y}-\underset{j\neq r}{\sum }a_{r,i}m_{j}. \end{array}$$

For more details on how we generate realizations from this truncated distribution, see [13].

### Generation of samples according to *f*(a_1:*R* − 1, *i*_|T, *σ*^**2**^, *R*, Y)

Straightforward computations lead to the posterior distribution of each element of **a**_1:*R* − 1, *i*_

(26) $$\begin{array}{ll} f\left(a_{1:R-1,i}|T,\sigma^{2},R,Y\right)\propto \exp & \left[-\frac{1}{2}{\bar{a}}_{1:R-1,i}^{T}\Sigma_{1:R-1,i}^{-1}{\bar{a}}_{1:R-1,i}\right]\\ & \times 1_{S}\left(a_{1:R-1,i}\right) \end{array}$$

where

(27) $$\begin{array}{ll} \;{\bar{a}}_{1:R-1,i} & =a_{1:R-1,i}-\mu_{1:R-1,i},\\ \;\Sigma_{1:R-1,i} & ={\left[{\bar{M}}_{\setminus R}^{T}\Sigma^{-1}{\bar{M}}_{\setminus R}\right]}^{-1},\\ \;\mu_{1:R-1,i} & =\Sigma_{1:R-1,i}\left[{\bar{M}}_{\setminus R}^{T}\Sigma^{-1}{\bar{M}}_{\setminus R}\right],\\ \;{\bar{M}}_{\setminus R} & =M_{\setminus R}-m_{R}1_{R-1}^{T}, \end{array}$$

$1_{R-1}=\left[1,\ldots ,1\right]\in R^{R-1}$ and **M**_∖*R*_ denotes the factor loading matrix **M** whose *R*-th column has been removed. Equation (26) shows that the factor score distribution is an MGD truncated on the simplex S defined in (12).

### Generation of samples according to *f*(*σ*^**2**^|T, A, *R*, Y)

Using (14) and (16), one can show that the conditional distribution *f*(*σ*^2^|**M**, **A**, **Y**) is the following inverse-Gamma distribution

(28) $$\sigma^{2}|M,A,Y∼IG\left(\frac{\text{GN}}{2},\frac{1}{2}\overset{N}{\underset{i=1}{\sum }}{∥y_{i}-{\mathrm{Ma}}_{i}∥}^{2}\right).$$

## Appendix B: Contribution of each of uBLU’s constraints

To illustrate the advantage of enforcing non-negativity and sum-to-one constraints on the factors and on the factor scores, as detailed in the Methods section, we evaluated the effect of successively stripping out these constraints from uBLU. In particular we implemented uBLU under the following conditions: i) without any constraints, ii) with only the positivity constraints on the factors and the scores, iii) with only the sum-to-one constraint on the scores, and iv) with both positivity and sum-to-one constraint on factors and scores as proposed in (5).

**Figure 6:**
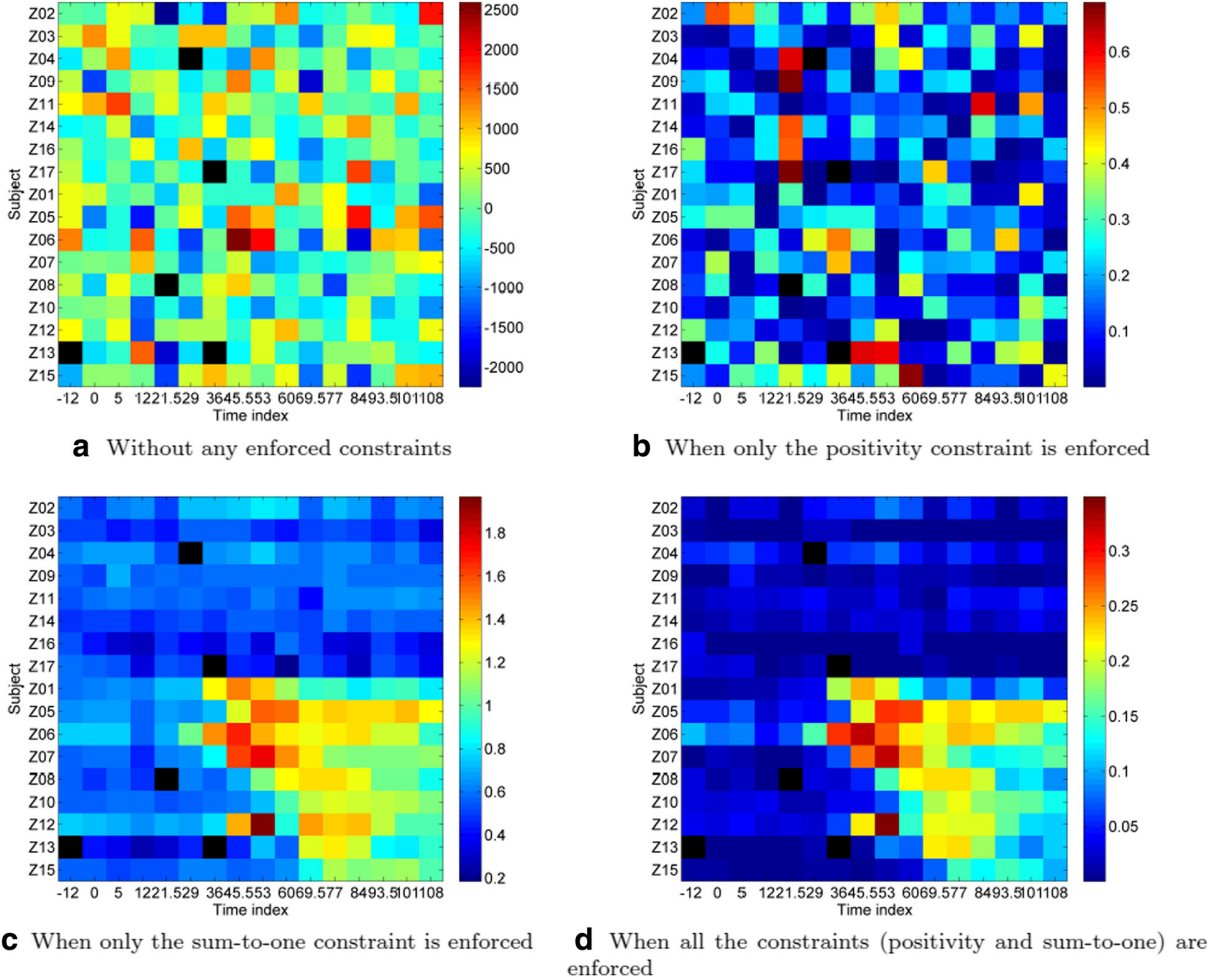
**Contribution of each constraint on the scores of the inflammatory factor (H3N2 challenge data).** The five black colored pixels of the heatmaps indicate samples that were not assayed. Note that when only the sum-to-one constraint is applied, non-negativity is not guaranteed. However, for this dataset the sum-to-one factor scores turn out to take on non-negative values for the inflammatory factor (but not for the other factors).

Figures 6 display heatmaps of the factor scores of the inflammatory component. The segmentation into two main regions (post-symptomatic samples and asymptomatic samples) becomes apparent only when the sum-to-one constraint is enforced on the scores. To quantify the benefit that is visible in Figure 6 we performed a GEA analysis, reported in Table 9, on the top 200 genes found in each of the inflammatory components found by uBLU implemented with no constraints, positivity constraints, sum-to-one constraints, and both constraints. The table shows that both constraints are necessary to obtain the best enrichment scores (lowest possible p-values).

**Table 9:** Contribution of each of uBLU’s constraints

	**Without**	**Positivity**	**Sum-to-one**	**Positivity and**
	**constraints**			**sum-to-one**
P-value of the “IFN-gamma pathway”	6.00.10^−2^	2.05.10^−2^	2.17.10^−1^	**1.34**.**10**^**−9**^
P-value of the “IL23-mediated signaling events”	2.60.10^−1^	8.37.10^−2^	2.28.10^−2^	**2.18**.**10**^**−7**^

Benefit of constraints in uBLU in terms of gene enrichment in the NCI-curated IFN-gamma and IL23-mediated pathways. As in Tables 6 and 8, the top 200 genes in the inflammatory components, whose scores are shown in Figures 6(a-d), were analyzed using the NCI Pathway Interaction Database. Both positivity and sum-to-one constraints are necessary for uBLU to reveal these two pathways with the high significance (p-value less than 10^−6^).

## Competing interests

The authors declare that they have no competing interests.

## Authors’ contributions

CB, ND, JYT and AH performed the statistical analysis. GG and AZ designed the Flu challenge experiment that generated the data used to compare the methods. All authors contributed to the manuscript and approved the final version.

## Supplementary Material

Additional file 1 **Supplementary materials on algorithm details and performance validation.** Directed acyclic graph (DAG) of the model and flowchart of the proposed algorithm are provided in this additional file. More results on synthetic datasets are also presented to validate the proposed Bayesian algorithm, including a convergence diagnosis. Click here for file
