# Supplementary material for: Unsupervised Bayesian linear unmixing of gene expression microarrays
Source: BMC Bioinformatics. 2013 Mar 19;14:99. doi: 10.1186/1471-2105-14-99 (PMC3681645; doi:10.1186/1471-2105-14-99)
Supplement: Additional file 1 — Supplementary materials on algorithm details and performance validation. Directed acyclic graph (DAG) of the model and flowchart of the proposed algorithm are provided in this additional file. More results on synthetic datasets are also presented to validate the proposed Bayesian algorithm, including a convergence diagnosis. [file 1471-2105-14-99-S1.pdf]

Unsupervised Bayesian linear unmixing of gene expression microarrays
Supplementary materials

Cécile Bazot[1], Nicolas Dobigeon[1], Jean-Yves Tourneret[1],
Aimee K. Zaas[2], Geoffrey S. Ginsburg[2], Alfred O. Hero III[3]

[1]Université de Toulouse, IRIT/INP-ENSEEIHT, Toulouse, France

[2]Duke University, Department of Medicine and Institute for Genome Sciences and Policy, Durham, USA

[3]University of Michigan, Center for Computational Biology and Bioinformatics and EECS Department, Ann Arbor, USA

{cecile.bazot, nicolas.dobigeon, jean-yves.tourneret}@enseeiht.fr,
{aimee.zaas, geoffrey.ginsburg}@duke.edu, hero@umich.edu

In this additional file, the directed acyclic graph (DAG) of the model and the flowchart of the proposed uBLU algorithm are provided. More results on synthetic datasets are presented to validate the proposed Bayesian algorithm.

1 Summary of the model

We consider the model described in the paper (see Section "Methods")

$$\mathbf{Y} = \mathbf{MA} + \mathbf{N} \tag{1}$$

or, for each sample ($i = 1, \ldots, N$)

$$\mathbf{y}_i = \sum_{r=1}^{R} \mathbf{m}_r a_{r,i} + \mathbf{n}_i = \mathbf{M}\mathbf{a}_i + \mathbf{n}_i \tag{2}$$

We propose to project the factors $\mathbf{m}_r$ ($r = 1, \ldots, R$) into a lower subspace. The factors and their corresponding projections $\mathbf{t}_r$ are related by

$$\mathbf{m}_r = \mathbf{P}^{-1}\mathbf{t}_r + \bar{\mathbf{y}}. \tag{3}$$

Moreover, the factor score vector $\mathbf{a}_i$ ($i = 1, \ldots, N$) can be rewritten as

$$\mathbf{a}_i = \begin{pmatrix} \mathbf{a}_{1:R-1,i} \\ a_{R,i} \end{pmatrix} \text{ with } \mathbf{a}_{1:R-1,i} = [a_{1,i}, \ldots, a_{R-1,i}]^T \tag{4}$$

and the last score $a_{R,i}$ is set to

$$a_{R,i} = 1 - \sum_{r=1}^{R-1} a_{r,i}.$$

The complete model and the priors chosen for the unknown parameters are defined by

$$\begin{aligned} \mathrm{P}[R = k] &= \frac{1}{R_{\max} - 1} \text{ for } R = 2, \ldots, R_{\max} & (5)\\ \mathbf{t}_r | \mathbf{e}_r, s_r^2 &\sim \mathcal{N}_{\mathcal{T}_r}\left(\mathbf{e}_r, s_r^2 \mathbf{I}_{R-1}\right) & (6)\\ \mathbf{a}_{1:R-1,i} | R &\sim \mathcal{U}_{\mathcal{S}}\left(\mathbf{a}_{1:R-1,i}\right) & (7)\\ \sigma^2 | \nu, \gamma &\sim \mathcal{IG}\left(\frac{\nu}{2}, \frac{\gamma}{2}\right). & (8) \end{aligned}$$

The resulting hierarchical structure of the different parameters and hyperparameters for the proposed uBLU model is summarized in the directed acyclic graph (DAG) shown in Figure 1. The fixed hyperparameters appear in dashed boxes. In our simulations, the mean vectors $\{\mathbf{e}_r\}_{r=1,\ldots,R}$ will be chosen as the PCA projections of the factors, previously identified by vertex component analysis (VCA) [1]. The variances $\{s_r^2\}_{r=1,\ldots,R}$ and the shape parameter ν will be respectively fixed to: $s_1^2 = \ldots = s_R^2 = 100$ and $\nu = 2$.

Figure 2 summarizes the proposed uBLU method. This diagram shows the two main steps of the proposed method: the first step updates the number of factors $R^{(t)}$ whereas the second one updates the matrices $\mathbf{M}^{(t)}$, $\mathbf{A}^{(t)}$ and the noise variance $\sigma^{2(t)}$ conditionally upon the updated number of factor $R^{(t)}$.

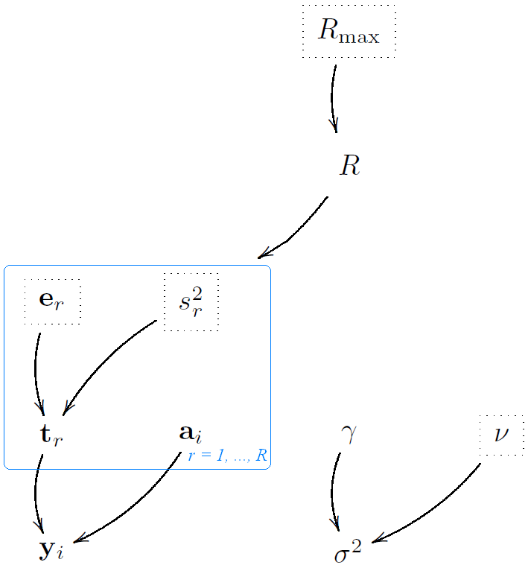

Fig. 1: **Directed acyclic graph (DAG) for the parameter priors and hyperpriors.**

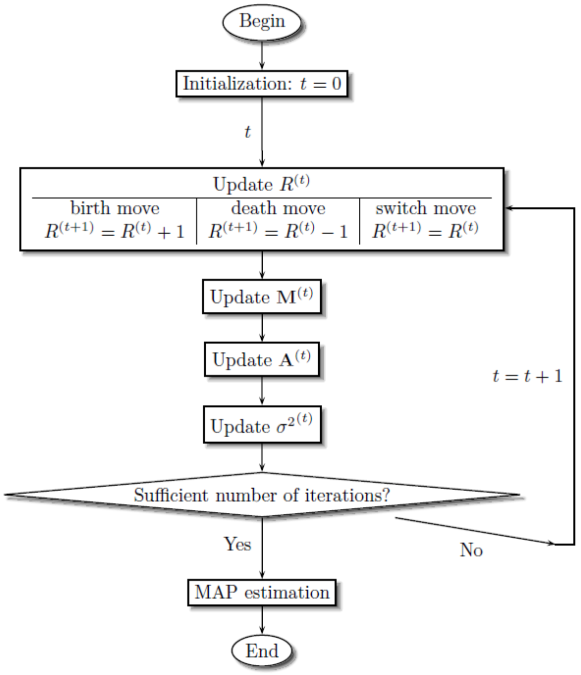

Fig. 2: **Flow diagram of the uBLU algorithm.**

2 Simulation scenario

The experiments presented in this file correspond to the expression value of $G = 512$ genes, for $N = 128$ samples. Each sample is composed of $R = 3$ different factors according to the linear mixing model (LMM) defined in (1) whose gene signatures are represented in Figure 3(a). The factors estimated by the proposed algorithm $[\hat{\mathbf{m}}_1, \hat{\mathbf{m}}_2, \hat{\mathbf{m}}_3]$ are in good agreement with the actual factors $[\mathbf{m}_1, \mathbf{m}_2, \mathbf{m}_3]$.

These $R = 3$ factors are mixed in random proportions (factor scores). The observations have been corrupted by an i.i.d. Gaussian noise sequence, with signal-to-noise ratio SNR = 20 dB where $\mathrm{SNR} = G^{-1}\sigma^{-2}\left\|\sum_{r=1}^{R}\mathbf{m}_r a_{r,i}\right\|^2$ for each sample i $(i = 1, \ldots, N)$.

The proposed algorithm has been run on synthetic data with $\mathrm{N}_{\mathrm{mc}} = 10000$ Monte Carlo iterations, including a burn-in period of $\mathrm{N}_{\mathrm{bi}} = 1000$ iterations.

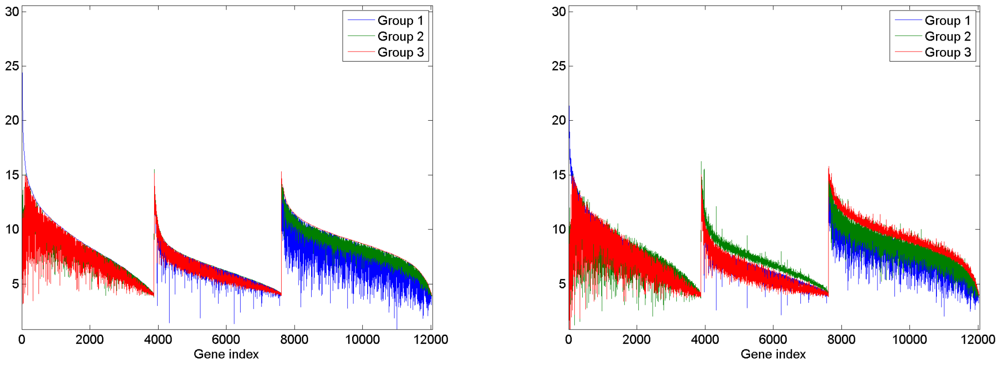

(a) Simulated factors (b) Estimated factors

FIG. 3: **Synthetic biological signatures (left) and their corresponding estimations (right).**

3 Convergence diagnosis

The Gibbs sampler, described in the Appendix, allows one to generate samples $\{\mathbf{M}^{(t)}, \mathbf{A}^{(t)}, \sigma^{2(t)}, R^{(t)}\}$ distributed according to the joint distribution $f\left(\mathbf{M}, \mathbf{A}, \sigma^2, R|\mathbf{Y}\right)$. The unknown parameters are then estimated using the generated samples. Here we assess convergence of the sampler and determine appropriate values for the number of MCMC iterations N_{mc} and the burn-in period N_{bi}.

3.1 Determination of the burn-in period $(\mathrm{N}_{\mathrm{bi}})$

For MCMC samplers, the most popular technique to assess convergence is due to Gelman and Rubin in [2]. The main idea is to run C parallel chains of same lengths N_{mc} initialized with over-dispersed values and then to calculate between- and within-variances. More recently, Brooks and Guidici [3] and Castelloe and Zimmerman [4] have extended Gelman and Rubin's technique to trans-dimensional samplers for which several models (values of R) can be selected. The convergence of the chain is then monitored by the following two criteria, related to the so-called *potential scale reduction factors* defined in [5]

$$\begin{aligned} PSRF_1 &= \frac{\widehat{V}(\theta)}{W_{\mathrm{c}}(\theta)}, \\ PSRF_2 &= \frac{W_{\mathrm{m}}(\theta)}{W_{\mathrm{m}}W_{\mathrm{c}}(\theta)}. \end{aligned} \tag{9}$$

where θ is the parameter of interest ($\theta \in \{\mathbf{M}, \mathbf{A}, \sigma^2\}$) and $\widehat{V}(\theta)$, $W_{\mathrm{c}}(\theta)$, $W_{\mathrm{m}}(\theta)$ and $W_{\mathrm{m}}W_{\mathrm{c}}(\theta)$ can be interpreted as total variation, variation within chains, variation within models and variation within models and chains, respectively. These parameters are defined in [4]. The two potential scale reduction factors $PSRF_1$ and $PSRF_2$ have been computed from $C = 10$ Markov chains for the parameter σ^2. For $\mathrm{N}_{\mathrm{bi}} = 5000$ iterations, the values of $PSRF_1$ and $PSRF_2$ are respectively equal to 0.93 and 1.04 for the synthetic dataset. These values confirm near optimal convergence of the sampler (close to the recommended values of 1 [4]).

3.2 Determination of the number of MCMC iterations ($\mathrm{N_{mc}}$)

An *ad hoc* approach is used to determine the appropriate number of MCMC iterations $\mathrm{N_{mc}}$ in order to obtain accurate estimators of the unknown parameters (see [6]. We compute and visualize the convergence of the reconstruction error $\mathrm{RE}^{(t)}$, plotted as a function of the iteration index ($t = 1, \ldots$)

$$\mathrm{RE}^{(t)} = \frac{1}{NG} \sum_{i=1}^{N} \|\mathbf{y}_i - \sum_{r=1}^{R^{(t)}} \mathbf{m}_r^{(t)} a_{r,i}^{(t)}\|^2. \tag{10}$$

Figure 4 shows the reconstruction error ($\mathrm{RE}^{(t)}$) and the estimated number of factors ($R^{(t)}$) as a function of the number of algorithm iterations (t) for the synthetic dataset and for two MCMC chains with two different starting points. This figure shows that a number of $\mathrm{N_{mc}} = 10000$ is sufficient to ensure accurate estimations of the unknown parameters for synthetic dataset, even if the model moves to another dimension (value of R) after the first 10000 iterations.

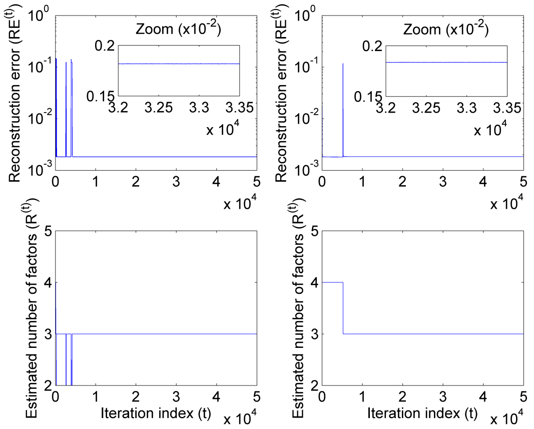

FIG. 4: **Convergence assessment on synthetic data for two different chains.** Top: Reconstruction error ($\mathrm{RE}^{(t)}$) computed from the observation matrix $\mathbf{Y}$ and the estimated matrices $\mathbf{M}^{(t)}$ and $\mathbf{A}^{(t)}$ at iteration t. Bottom: Corresponding estimated number of factors $R^{(t)}$ at iteration t.

4 Results on synthetic datasets

The first step of the algorithm consists of estimating the number of factors R involved in the mixture, and hence determining the dimensions of the matrices $\mathbf{M}$ and $\mathbf{A}$, using the *maximum a posteriori* (MAP) estimator $\hat{R}_{\mathrm{MAP}}$. The estimated posterior distribution of R depicted in Figure 5(a) is clearly in agreement with the actual value of R: the MAP estimator is $\hat{R}_{\mathrm{MAP}} = 3$. This figure also shows that the proposed algorithm moves between spaces with different dimensions (corresponding to $R = 2$, $R = 3$, and $R = 4$).

The second step of the algorithm consists of estimating the unknown model parameters ($\mathbf{M}$, $\mathbf{A}$ and σ^2) conditionally upon $\hat{R}_{\mathrm{MAP}}$. The factor signatures estimated by the proposed algorithm are represented in Figure **??**. The estimated posterior distributions of the factor scores obtained for the particular sample ♯30 are depicted in Figure 5(b). These estimated posterior distributions have been computed from $M = 20$ Markov chains, i.e., 20 noise realizations on the same synthetic dataset. These posteriors are clearly in good agreement with the actual values of the factor scores depicted in red lines.

Figure 4 shows the reconstruction error ($\mathrm{RE}^{(t)}$) as a function of the number of algorithm iterations (t) for two MCMC chains on the synthetic dataset. This figure shows that different moves (birth, death or switch moves) are accepted by the proposed algorithm.

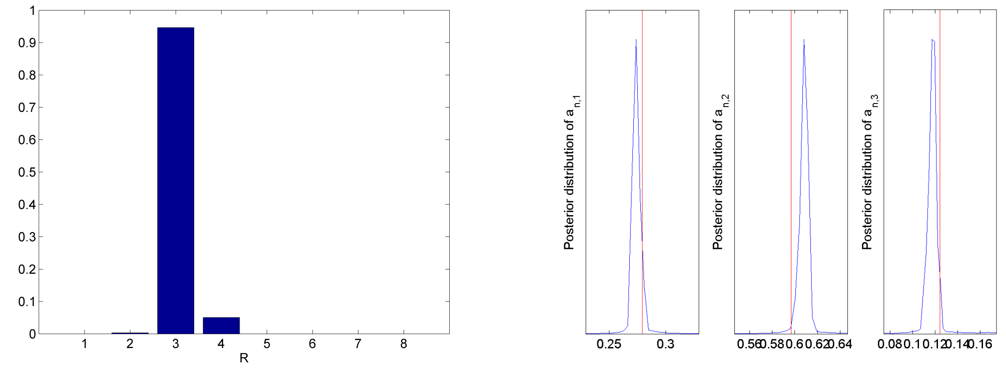

(a) Estimated posterior probability for the number of factors

(b) Estimated posterior of the factor scores

FIG. 5: **Estimated posteriors (synthetic data).** Estimated posterior distributions for the number of factors R involved in the mixture (a) and the factor scores $[a_{i,1}, a_{i,2}, a_{i,3}]^T$ conditionally upon the estimated number of factors $\widehat{R}_{\text{MAP}} = 3$ (b).

References

[1] Nascimento JM, Bioucas-Dias JM: **Vertex component analysis: A fast algorithm to unmix hyperspectral data**. *IEEE Trans. Geosci. and Remote Sensing* 2005, **43**(4):898–910.

[2] Gelman A, Rubin DB: **Inference from iterative simulation using multiple sequences**. *Statistical Science* 1992, **7**:457–511.

[3] Brooks SP, Roberts GO: **Assessing Convergence of Markov Chain Monte Carlo Algorithms**. *Statistics and Computing* 1997, **8**:319–335.

[4] Castelloe JM, Zimmerman DL: **Convergence Assessment for Reversible Jump MCMC Samplers**. Tech. rep. 2002.

[5] Gelman A, Carlin JB, Stern HS, Rubin DB: *Bayesian Data Analysis, Second Edition (Chapman & Hall/CRC Texts in Statistical Science)*. London: Chapman and Hall/CRC, 2 edition 2003.

[6] Dobigeon N, Tourneret JY, Chang CI: **Semi-supervised linear spectral unmixing using a hierarchical Bayesian model for hyperspectral imagery**. *IEEE Trans. Signal Processing* 2008, **56**(7):2684–2695.
